# Oncogenic Integration of Nucleotide Metabolism *via* Fatty Acid Synthase in Non-Hodgkin Lymphoma

**DOI:** 10.3389/fonc.2021.725137

**Published:** 2021-10-26

**Authors:** Dashnamoorthy Ravi, Afshin Beheshti, Nasséra Abermil, Frederick Lansigan, William Kinlaw, Nirupa R. Matthan, Maisarah Mokhtar, Frank C. Passero, Patrick Puliti, Kevin A. David, Gregory G. Dolnikowski, Xiaoyang Su, Ying Chen, Mahboubi Bijan, Rohan R. Varshney, Baek Kim, Sandeep S. Dave, Michael C. Rudolph, Andrew M. Evens

**Affiliations:** ^1^ Division of Blood Disorders, Rutgers Cancer Institute of New Jersey, New Brunswick, NJ, United States; ^2^ Department of Medicine, Robert Wood Johnson Medical School, Rutgers University, New Brunswick, NJ, United States; ^3^ Stanley Center for Psychiatric Research, Broad Institute of Massachusetts Institute of Technology and Harvard, Cambridge, MA, United States; ^4^ KBR, Space Biosciences Division, National Aeronautical and Space Administration, Ames Research Center, Moffett Field, CA, United States; ^5^ Assistance Publique-Hôpitaux de Paris (AP-HP), Hôpital Saint-Antoine, Service d’Hématologie Biologique, Paris, France; ^6^ Department of Medicine, Norris Cotton Cancer Center, Dartmouth-Hitchcock Medical Center, Lebanon, NH, United States; ^7^ Department of Medicine, Section of Endocrinology and Metabolism, Geisel School of Medicine at Dartmouth, Hanover, NH, United States; ^8^ Department of Medicine, Norris Cotton Cancer Center, Geisel School of Medicine at Dartmouth, Lebanon, NH, United States; ^9^ Jean Mayer United States Department of Agriculture (USDA) Human Nutrition Research Center on Aging, Tufts University, Boston, MA, United States; ^10^ Department of Medicine, University of Rochester Medical Center, Rochester, NY, United States; ^11^ Metabolomics Core, Rutgers Cancer Institute of New Jersey, New Brunswick, NJ, United States; ^12^ Bioinformatics Core, Rutgers Cancer Institute of New Jersey, New Brunswick, NJ, United States; ^13^ Department of Pediatrics, School of Medicine, Emory University, Atlanta, GA, United States; ^14^ Harold Hamm Diabetes Center, The University of Oklahoma Health Sciences Center, Oklahoma, OK, United States; ^15^ Center for Drug Discovery, Children’s Healthcare of Atlanta, Atlanta, GA, United States; ^16^ Department of Medicine, Duke Cancer Institute, Duke University Medical Center, Durham, NC, United States

**Keywords:** non-Hodgkin lymphoma, FASN, metabolomics, nucleotides, pentose phosphate pathway, lipid metabolism

## Abstract

Metabolic dysfunctions enabling increased nucleotide biosynthesis are necessary for supporting malignant proliferation. Our investigations indicate that upregulation of fatty acid synthase (FASN) and *de novo* lipogenesis, commonly observed in many cancers, are associated with nucleotide metabolic dysfunction in lymphoma. The results from our experiments showed that ribonucleotide and deoxyribonucleotide pool depletion, suppression of global RNA/DNA synthesis, and cell cycle inhibition occurred in the presence of FASN inhibition. Subsequently, we observed that FASN inhibition caused metabolic blockade in the rate-limiting step of the oxidative branch of the pentose phosphate pathway (oxPPP) catalyzed by phosphogluconate dehydrogenase (PGDH). Furthermore, we determined that FASN inhibitor treatment resulted in NADPH accumulation and inhibition of PGDH enzyme activity. NADPH is a cofactor utilized by FASN, also a known allosteric inhibitor of PGDH. Through cell-free enzyme assays consisting of FASN and PGDH, we delineated that the PGDH-catalyzed ribulose-5-phosphate synthesis is enhanced in the presence of FASN and is suppressed by increasing concentrations of NADPH. Additionally, we observed that FASN and PGDH were colocalized in the cytosol. The results from these experiments led us to conclude that NADP–NADPH turnover and the reciprocal stimulation of FASN and PGDH catalysis are involved in promoting oxPPP and nucleotide biosynthesis in lymphoma. Finally, a transcriptomic analysis of non-Hodgkin’s lymphoma (*n* = 624) revealed the increased expression of genes associated with metabolic functions interlinked with oxPPP, while the expression of genes participating in oxPPP remained unaltered. Together we conclude that FASN–PGDH enzymatic interactions are involved in enabling oxPPP and nucleotide metabolic dysfunction in lymphoma tumors.

## Introduction

Oncogenic *de novo* lipogenesis, which is catalyzed by the overexpressed fatty acid synthase (FASN), is an important metabolic phenotype observed in many cancers (1). FASN is a 273-kDa cytosolic multi-catalytic enzyme complex consisting of homo-dimeric subunits with head to tail linked configuration, which catalyzes the biosynthesis of palmitic acid (2). FASN activity is entirely dependent on cytosolic glucose metabolism for the substrates acetyl-CoA and malonyl-CoA, both derived through glycolysis/citric acid cycle, and coenzyme NADPH, derived from the pentose phosphate pathway (PPP) (2), as indicated in the following reaction equation.


$$\text{Acetyl}-\text{CoA}+7\text{malonyl}-\text{CoA}+14\text{NADPH}+14H^{+}\to \text{palmitic acid}+8\text{CoA}-\text{SH}+7CO_{2}+6H_{2}O+14\text{NAD}P^{+}$$

While other enzymatic sources of cytosolic NADPH are known to exist, PPP is regarded as the primary NADPH source for human FASN activity (3). Palmitic acid, which is the end-product in FASN enzyme activity, is also a key intermediate for lipid metabolism. Palmitic acid is utilized in the biosynthesis of phospholipids, sphingolipids, ether lipids, diacylglycerol, and ceramide. Most importantly, several palmitic acid-derived lipids act as second messengers and are involved in the regulation of growth and immune-related signaling pathways, including, PI3K, MAPK, and NFκB (2, 4–6). Moreover, palmitic acid, through protein palmitoylation, is known to impact receptor aggregation and protein mobilization dynamics on the cell surface (7, 8). In premalignant cells, FASN activity induced by HIF1α is associated with restoring glycolysis and oxidative phosphorylation from hypoxia-induced metabolic suppression (9). In malignant cells, FASN upregulation mediated by oncogenic signals (including HER2, EGFR, MAPK, and PI3K) through sterol response element binding proteins is surmised as lipogenic in nature (2).

We have previously reported the occurrence of PI3K alterations (10) and increased FASN expression in non-Hodgkin lymphoma (NHL) (11). Furthermore, we also observed that an increased FASN expression was correlated with a poor clinical outcome in NHL (11). Collectively, there remains a desire to identify novel targeted therapeutic options with better efficacy and relatively fewer toxic profiles for the treatment of NHL (12, 13). Therefore, we evaluated the potency of several FASN small molecule inhibitors, including cerulenin, orlistat, TVB3166, and TVB3567, in NHL experimental models. Cerulenin is an antifungal antibiotic FASN inhibitor, which binds irreversibly with the catalytic domain of β-keto acyl synthase subunit and blocks the initial step of FASN-catalyzed condensation of acetyl-CoA with malonyl-CoA (14). The anticancer activity of cerulenin-mediated FASN inhibition has been extensively investigated using multiple *in vivo* and *in vitro* tumor models (5). Similarly, orlistat, a bispecific FASN and pancreatic lipase inhibitor, and the novel small-molecule FASN inhibitors, TVB3166 and TVB3567, have been evaluated in several solid tumor models (15, 16). In the present study, we define the biological consequences and significance of FASN inhibition in NHL.

## Methods

### Cell Culture, Reagents, And Transfections

ATCC (STR profiling) authenticated bNHL cells, SUDHL2, SUDHL4, SUDHL10, and OCI-LY19. Raji was grown in the RPMI 1640 medium with 10% heat-inactivated fetal bovine serum (FBS) and 200 U of penicillin/streptomycin (Mediatech, Manassas, VA) under 5% CO_2_ and at 37°C. Primary DLBCL tumor cells were obtained through Tufts Tumor Repository at Tufts Medical Center (Boston, MA) as de-identified discarded specimens through an exempt institutional review board approval. The FASN inhibitors cerulenin (Sigma Aldrich, St. Louis, MO), TVB3166, and TVB3567 were generous gifts from 3V Biosciences (Menlo Park, CA). Standards from quantitative mass spectrometry nucleotides, 6-phosphogluconate and glucose-6-phosphate, were purchased from Sigma (St. Louis, MO). RNA interference experiments were performed using FASN siRNA Ambion Cat# 4390824 (Thermo Fisher Scientific, Austin, TX) and PGDH shRNA, Mission shRNA clone TRCN0000274974 (Sigma Aldrich, St. Louis, MO).

### Western Blot

We prepared the protein lysates and performed western blots as described before (17) using the following primary antibodies against total and cleaved caspase-3 and PARP. PGDH, FASN, and β-actin were purchased from Cell Signaling Technology (Beverly, MA).

### Cell Viability Assays

MTT assays were performed using bNHL cells treated with cerulenin for 72 h, as described before (17). IC_50_ values for drug treatments were derived using Calcusyn version 2.1 software (Biosoft, Ferguson, MO).

### Flow Cytometry

Apoptosis was determined using annexin-V/propidium iodide (PI) staining and flow cytometry, as described before (17).

### Transcriptome Analysis

RNA isolation and transcriptomic analysis by gene set enrichment analysis (GSEA) and ingenuity pathway analysis (IPA) were performed as described before (17–19). All experiments were performed in biological triplicates. Affymetrix Human Genechip 2.0 ST was used for cerulenin-treated Raji and SUDHL10, and Human HT 12 Genechip Illumina was used for cerulenin-, TVB3166-, or TVB3567-treated Raji, SUDHL2, SUDHL10, or SUDHL4 cells. The raw data from these experiments is available at the NCBI Gene Expression Omnibus database, with the following identifiers: GSE102760 for cerulenin-treated Raji and SUDHL10 experiments and GSE102764 for cerulenin-treated SUDHL4 experiments and for cerulenin-, TVB3166-, or TVB3567-treated Raji, SUDHL2 SUDHL10 experiments. Previously published RNA seq data set available from 624 NHL patients (20) were utilized for metabolic gene expression. Lists of metabolic genes were downloaded from Wikipathways, Reactome, and KEGG databases, and a curated gene list was used for gene expression analysis and construction of metabolic pathway models and heatmap by R package (ComplexHeatmap), as described previously (21).

### Metabolic Profiling

bNHL cells cultured in the presence of D-glucose-^13^C_6_ 2 g/L (Cambridge Isotopes, Tewksbury, MA) in glucose-free RPMI-1640 (Sigma Aldrich, St. Louis, MO) containing 10% FBS were treated with an appropriate concentration of drugs for 48 h. Lipids were extracted using a modified Folch method (22), followed by saponification using 0.5 N methanolic sodium hydroxide and methylated by boron trifluoride in methanol as described previously (23). The supernatant containing the fatty acid methyl esters was dried under nitrogen and resuspended in acetonitrile for lipid profiling by liquid chromatography–mass spectrometry (LC−MS). For polar metabolites, samples extracted with (40:40:10) methanol, acetonitrile, and water, consisting of 0.5% formic acid and neutralized with sodium bicarbonate, were used for analysis by mass spectrometry. LC−MS was performed using the Q Exactive PLUS hybrid quadrupole-orbitrap mass spectrometer (Thermo Scientific) coupled to hydrophilic interaction chromatography. Metabolite features were extracted using MAVEN with labeled isotope specified and a mass accuracy window of 5 ppm (24). The ^13^C isotope natural abundance and the impurity of labeled substrate were corrected using AccuCor written in R as described (25). The corrected ion counts were normalized by cell number. The processed datasets were statistically analyzed, and feature identification by principal component analysis (PCA), partial least squares-discriminant analysis (PLS-DA), PatternHunter, correlation clustering and heat map analysis, and joint pathway analysis were performed using Metaboanalyst 3.0 software (26, 27).

### dNTP Assays

Cellular dNTP levels were determined using a RT-based dNTP assay, as described previously (28).

### Global DNA and RNA Synthesis

DNA synthesis was monitored using EZClick EdU cell proliferation kit (#K946). Confocal microscopy and RNA synthesis, quantified by flow cytometry, were performed using 5-ethynyl-uridine-based EZClick Global RNA synthesis assay kit (#K718) purchased from Biovision (Milpitas, CA), following the instructions supplied by the manufacturer.

### NADP/NADPH Assay

Total NADP/NADPH concentration was determined using NADP/NADPH-Glo assay (Promega, Madison, WI), following the manufacturer-supplied instructions, using NHL cells treated with cerulenin for 48 h.

### Enzyme Activity Assays

Enzymatic activity assays were performed using the following kit purchased from Abcam (Cambridge, MA): phospho-gluconate dehydrogenase (PGDH) #ab155896. The assays were performed following the manufacturer-supplied instructions.

### FASN PGDH Colocalization Analysis

Cytospin preparation consisting of 1 × 10^5^ cells was performed using EZ Cytofunnel starter kit and Cytospin 4 (Thermo Scientific, Waltham, MA). Air-dried and methanol-fixed cell preparations were permeabilized with 0.25% Triton X-100 in phosphate-buffered saline, blocked using 1% bovine serum albumin, and incubated with appropriate antibody concentrations. Stained cells mounted using Prolong Antifade-Gold reagent (Molecular Probes, by Thermo Fisher Scientific, Waltham, MA) were used for image acquisition by a Nikon A1RSi laser confocal microscope. Colocalization analysis was performed using Colocalization and JACoP, plugins available through ImageJ, as described before (29). Primary antibodies mouse anti-human FASN antibody clone 3F2-1F3 (LSBio, LifeSpan, cat. #LS-C104946, Seattle, WA) and rabbit anti-human PGDH (Cell Signaling Technology, cat. #13389, Danvers, MA) and the following secondary antibodies, AlexaFluor-594 goat and anti-mouse and AlexaFluor-488 donkey anti-rabbit (Invitrogen), were used in this study.

### Cell-Free Enzyme Assays

The human recombinant PGDH was purchased from ProSpec Bio (East Brunswick, NJ). The human recombinant FASN is a generous gift from Dr. Michael C. Rudolph. The preparation, purification, reconstitution, and activity assessments are described elsewhere (30, 31). The following substrates and coenzyme factors for cell-free enzyme assays were purchased from Sigma Aldrich (St. Louis, MO): 6-phosphogluconate (6PG), acetyl-CoA, malonyl-CoA, β-NADP, and β-NADPH. The reactions were performed using 250 mM potassium phosphate buffer, pH 7.6, consisting of 1 mM DTT and 5 μM EDTA. The reactions were performed using PGDH (0.25 μg), FASN (4 μg), 2 mM 6PG, 40 μM acetyl-CoA, 110 μM malonyl-CoA, and variable concentrations of NADP or NADPH (0–800 μM) in 100-μl final volume. The reaction kinetics were monitored continuously for 20 min at 340 nM, for NADPH appearance or disappearance, using 96-well half area U-plate (Costar) and Tecan infinite M200 plate reader. The reactions were quenched by the addition of ice-cold 1:1 methanol and acetonitrile consisting of 0.5% formic acid, and the mixture was incubated on ice for 5 min, neutralized with sodium bicarbonate, and centrifuged. The collected supernatants were used for mass spectrometric analysis.

### Statistical Analysis

All experiments were performed in triplicate. Significant differences between control and treatment were statistically determined by Student’s *t*-test for cell viability, apoptosis, and enzyme activity assays. For metabolic profiling experiments, identification of top significant metabolite features by PLS-DA and variable importance in projection (VIP) scoring analysis, statistical correlation analysis by one-way ANOVA, and *post-hoc* analysis and Spearman rank correlation were performed using the software packages included in Metaboanalyst 3.0 (26, 27). The statistical analysis for transcriptomic datasets was performed as previously described (17–19).

## Results

### FASN Inhibition Induces Cell Death in bNHL

**Figure 1:**
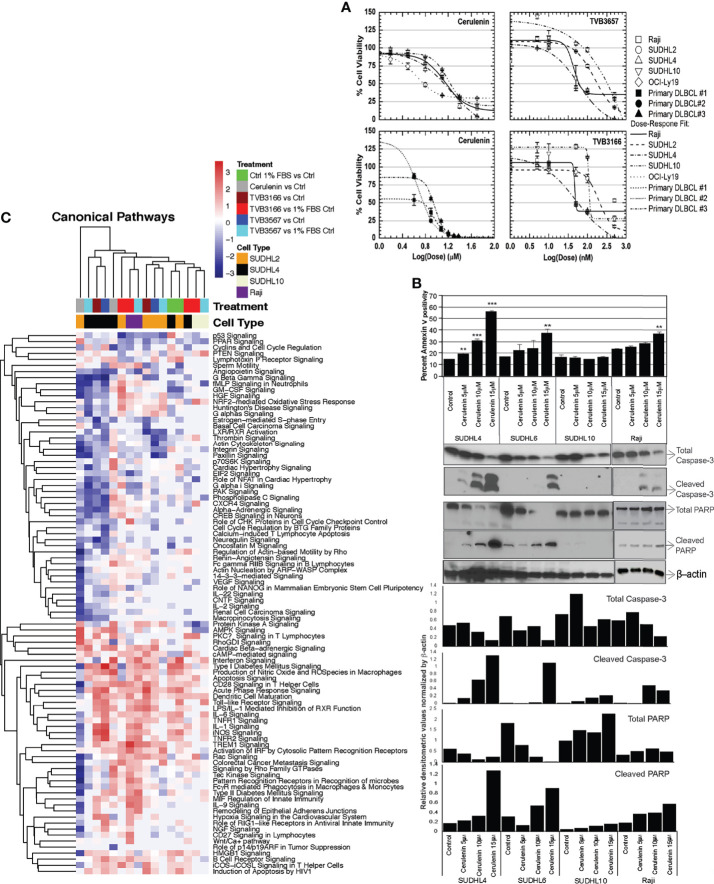
Cell viability, apoptosis, and transcriptomic analysis with FASN inhibitors in bNHL. **(A)** Dose–response curves from cerulenin-, TVB3166-, and TVB3567-treated bNHL cell lines and patient-derived primary bNHL (DLBCL) cells, with concentration in (x-axis) and dependent decrease in percent cell viability plotted in (y-axis), determined at 72 h by MTT assay with cerulenin or at 7 days by CellTiter-Glo assay with TVB3166 and TVB3567. **(B)** Bar chart representing annexin-v staining positivity (y-axis) with cerulenin concentration by individual bNHL cells on (x-axis), determined by flow cytometry, along with corresponding western blot analysis for apoptosis markers, showing the activation of caspases and PARP cleavage, below. The error bars represent the standard deviations of mean, and the significant difference between control and treatment is indicated by an asterisk (**p < 0.05 and ***p < 0.005) statistically determined by Student’s *t*-test. The bar graph below the western blots represents the ratio of caspase-3 or PARP products normalized by total β-actin. **(C)** Hierarchical clustering showing the canonical pathway changes as a heat map of Z scores representing activation or inhibition occurring with cerulenin or TVB3166 and TVB3567 treatment in lymphoma cells.

Treatment with increasing concentrations of cerulenin for 72 h resulted in a dose-dependent reduction in cell viability with associated induction of apoptosis in bNHL cells (**Figure 1**). The corresponding IC_50_ values for the cerulenin treatment were as follows: Raji (14.3 μM), SUDHL4 (8.0 μM), SUDHL10 (19.4 μM), and OCI-LY19 (9.6 μM) (**Figure 1A**). In primary bNHL tumor cells, the associated IC_50_ values were 5.1 μM in DLBCL #1, 4.04 μM in DLBCL #2, and 7.8 μM in DLBCL #3 (**Figure 1A**). We also noted that the sensitivity of DLBCL cells was significantly low with IC_50_ >50 μM for orlistat compared with cerulenin (data not shown). The IC_50_ values of novel small-molecule FASN inhibitors TVB3166 or TVB3156, respectively, were as follows: Raji—110 and 115 nM, SUDHL2—227 and 222 nM, SUDHL4—78 and 85 nM, and SUDHL10—433 and 863 nM (**Figure 1A**). The annexin-V-based flow cytometry showed a dose-dependent increase in apoptosis in SUDHL4 and Raji, but not in SUDHL10 cells (**Figure 1B**). These results were confirmed by western blot analysis for markers of apoptosis, which showed the presence of cleaved caspase 3 and PARP in the cerulenin-treated bNHL (**Figure 1B**), except in cerulenin-treated SUDHL10, indicating resistance to FASN inhibition in these cells.

### FASN Inhibitory Transcriptome

The global transcriptomic analyses following cerulenin treatment showed significant differential gene changes for DLBCL cells SUDHL2 (141 genes), SUDHL4 (242 genes), and SUDHL10 (1,600 genes) and Raji (Burkitt lymphoma) (1,092 genes) (**Supplementary Figures S1A–D, G**). Inhibition of FASN by TVB3166 or TVB3567 showed differentially expressed significant genes for SUDHL2 (962 and 600 genes), SUDHL10 (779 and 497 genes), and Raji (2,216 and 2,556 genes) (**Supplementary Figures S1E–G**). The canonical pathway analysis by IPA of these FASN inhibitory transcriptomes identified a conserved upregulation of immune signaling (tumor necrosis factor, interferon, CD27, CD28, IL1, IL6, IL9, and BCR signaling), apoptosis signaling, cAMP, protein kinase A, and NRF-2-mediated oxidative stress response pathways as activated mechanisms by FASN inhibition in NHL cells (**Figure 1C**). Genes down-regulated by FASN inhibition included cell cycle functions (mediated by cyclins, estrogen, and checkpoint proteins) and growth regulation (mediated by p53, PPAR, phospholipase C, p70 S6K, VEGF, IL2, IL22, GM-CSF, and HGF) as identified by the IPA analysis of all NHL cells (**Figure 1C**). The transcriptional network analysis from GSEA showed nucleotide/RNA metabolism and cell cycle as down-regulated mechanisms from cerulenin or TVB3166 and TVB3567 treatment in NHL cells (**Supplementary Figure S2**). Taken together, the gene expression analyses by IPA and GSEA both indicate that cell cycle and its regulatory functions are negatively impacted by FASN inhibition.

### FASN Inhibitory Metabolome

**Figure 2:**
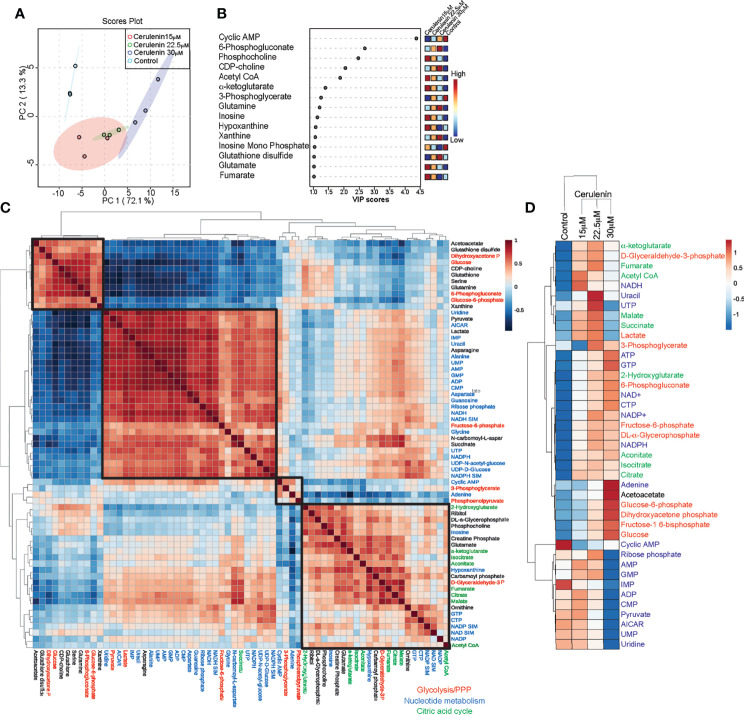
Metabolic impacts of cerulenin-mediated FASN inhibition in SUDHL10 cells. **(A)** Score plot from SUDHL10 cells by principal component analysis (PCA) indicates variances (shown within brackets) to metabolic profiles resulting from cerulenin treatment. **(B)** Identification of top significant metabolite features (*P* < 0.001) which contributed to differential PCA loading by partial least squares—discriminant analysis and variable importance in projection scoring analysis. The colored boxes on the right indicate the relative concentrations of the corresponding metabolite in each group. **(C)** Correlation heat map comparing the top 50 significant features with *p*-value and false discovery rate <0.05 determined by one-way ANOVA and *post-hoc* analysis, identified distinct patterns of correlative associations (distance measured by Spearman rank correlation) between metabolites affected by cerulenin treatment in SUDHL10 cells. In the correlation matrix, a positive correlation coefficient between concordant metabolites is represented in red, and a negative correlation coefficient between discordant metabolites is represented as blue. Legend depicting the metabolites shown in colors represents the following: red—glucose metabolism, blue—nucleotide metabolism, and green—citric acid cycle. **(D)** Heat map representing hierarchical clustering by correlation distance and average linkage of log_2_-transformed; row-centered data show cerulenin dose effect on individual metabolite in experimental triplicates, indicating either increased (in red) or decreased (in blue) pool sizes observed with FASN inhibition in SUDHL10 cells.

We next performed metabolic profiling to determine the impact of FASN inhibition on cellular metabolism. For this, SUDHL10 cells treated with increasing concentrations of cerulenin for 48 h were utilized for metabolic assessments by mass spectrometry. The PCA score plot comparing the cerulenin (15–30 μM) treatment indicated 72% variance by differential loading across the PC1 axis in metabolic alterations compared to untreated SUDHL10 cells (**Figure 2A**). The regression-based PLS-DA of the top 50 metabolic features identified from one-way ANOVA analysis resulted in the selection of 15 metabolites. These identified 15 features were then ranked by VIP scoring analysis with significant *P*-values <0.001, which identified cAMP, 6PG, phosphocholine, CDP-choline, and acetyl-CoA among other metabolites **(****Figure 2B**). The heat map of this plot showed that the levels of 6-phosphogluconate, CDP-choline, and acetyl-CoA were increased with cerulenin treatment in a concentration-dependent manner **(****Figure 2B**). The correlation matrix analysis comparing the cerulenin dose effect with the responses of significant metabolites revealed four major clusters across the diagonal within the heat map (**Figure 2C**). The first cluster (group 1) consisted of acetoacetate (acetyl-CoA-derived ketone body), reduced and oxidized glutathione, glucose, glucose-6-phosphate, and 6PG (PPP metabolites), CDP-choline, and amino acids (serine and glutamine) (**Figure 2C**). The second cluster (group 2) in the middle section included metabolites predominantly representing nucleotide metabolism (uridine, uracil, nucleotide monophosphates, ribose + ribulose-5-phosphate (R5P), AICAR, coenzymes—reduced (NADH/NADPH), alanine, aspartate, and glycine amino acids related to the *de novo* biosynthesis of nucleotides), and glycolytic intermediates (fructose-6-phosphate, pyruvate, and lactate) (**Figure 2C**). The third minor cluster (group 3) in the middle mid-segment consisted of cAMP, adenine, 3-phosphoglycerate, and phosphoenolpyruvate, representing both nucleotide and glucose metabolism (**Figure 2C**). The fourth cluster (group 4) in the right lower corner of the heat map consisted of metabolites mostly representing the citric acid cycle, and this cluster included FASN substrate and acetyl-CoA (**Figure 2C**). Comparing the group 1 metabolites with the metabolites from groups 2–4, we observed that a strong negative correlation exists between these sets. Interestingly, group 2 metabolites, predominantly comprised of nucleotide metabolites, exhibited a strong negative correlation with group 1, which consisted of metabolites representing the interrelated PPP metabolism. Now, comparing the absolute metabolic changes represented as heat map, we observed that cerulenin treatment resulted in increased levels of metabolites of pathways (glycolysis, PPP and citric acid cycle) that feed into the synthesis of FASN substrate acetyl CoA and NADPH generation, while a reduction in nucleotide pools relevant to *de novo* biosynthesis (IMP, AICAR, AMP, GMP, CMP, and UMP) was observed with FASN inhibition (**Figure 2D**).

### FASN Inhibition Impacts Nucleotide Metabolism

**Figure 3:**
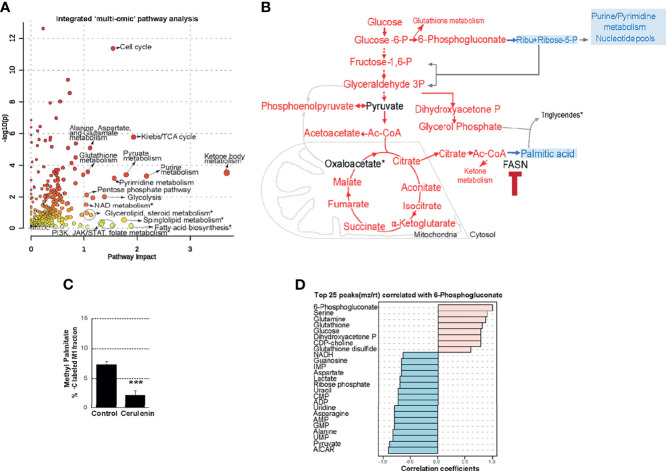
Effects of FASN inhibition on PPP metabolism. **(A)** Scatter plot representing enriched pathways identified by integrated multi-omic analysis of cerulenin transcriptome and metabolome, with *P*-values plotted as -log_10_ in y-axis and impact scores on x-axis. Top 20 high impact pathways with impact scores >1, FDR, and *P*-value cutoff <0.5 are indicated by names. Asterisk denotes non-significant high-impact pathways below the FDR and *P*-value cutoff. The complete list of pathways with statistical values from this analysis is included in **Supplementary Table S1**. **(B)** The summary of metabolic changes shown in this flow chart indicates the levels of metabolites that “increased” (denoted in red) or “decreased” (denoted in blue) by cerulenin treatment in SUDHL10 cells. **(C)** Bar graph representing the percentage of C^13^ labeling (x-axis) in the M1 peak of methyl palmitate fractions (y-axis) comparing the control with cerulenin treatment in SUDHL10 cells. Data represented in the bar graph represent averages from experimental triplicates, comparing cerulenin-treated cells with untreated control with *P*-values <0.001 denoted as ***. **(D)** Identification of top 25 metabolites (y-axis) correlating with 6-phosphogluconate level changes associated with FASN inhibition in SUDHL10 cells by Spearman rank correlation and distance measured by PatternHunter.

We then fused transcriptomic and metabolomic datasets and performed topological assessment of centrality and determination of pathway enrichment scores, using joint-pathway analysis by Metaboanalyst. This process resulted in the identification of ketone body metabolism, Krebs (citric acid cycle), nucleotide metabolism (purine/pyrimidine metabolism), cell cycle, amino acid and glutathione metabolism, and PPP and NAD metabolism as the most FASN inhibition-impacted pathways with enrichment scores >1, false discovery rate (FDR), and *P*-values <0.05 (**Figure 3A** and **Supplementary Table S1**). Other high-impact pathways that included several lipid metabolic processes and PI3K/JAK/STAT signaling pathways were identified but were below the cutoff for statistical stringency (**Figure 3A** and **Supplementary Table S1**). In summarizing these observations, FASN inhibition resulted in increasing the levels of metabolic intermediates associated with glycolysis and citric acid cycle that yield acetyl-CoA as substrate for FASN activity (indicated in red) (**Figure 3B**). The inhibition of FASN is expected to interrupt palmitic acid synthesis; towards this end, we observed decreased palmitic acid synthesis (determined based on C^13^ fractional labeling) that occurred with cerulenin treatment in SUDHL10 (**Figure 3C**). Thus, with acetyl-CoA accumulation and decreased palmitic acid synthesis, we also observed pool size reductions in R5P and nucleotides (denoted in blue) occurring with FASN inhibition (**Figure 3B****)**. Biochemically, oxidative PPP supplies NADPH for FASN. Thus, the accumulation of 6-phosphogluconate (6PG) (denoted in red) and the reduction in R5P (denoted in blue) (**Figure 3B**) suggest the perturbation of PGDH-catalyzed PPP activity occurring with FASN inhibition.

**Figure 4:**
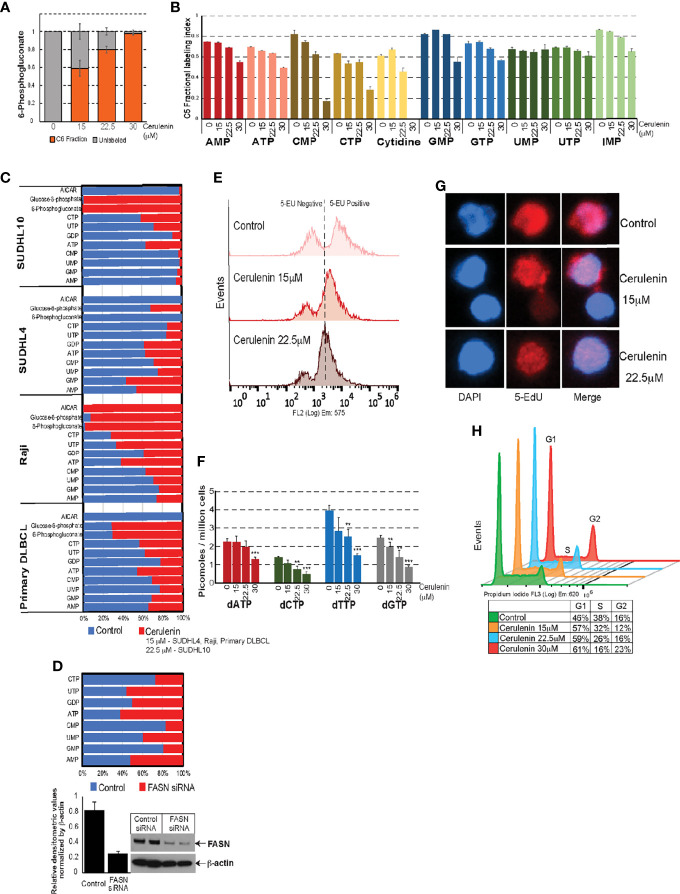
Effects of FASN inhibition on nucleotide metabolism. **(A, B)** Bar graphs representing C^13^ fractional incorporation (in y-axis) in C5 residues of 6PG or nucleotide pools listed on the x-axis, with cerulenin-treated SUDHL10 cells. **(C, D)** Quantitative mass spectrometry analysis of nucleotide pools represented as fold change (by percentage) with the levels of nucleotides occurring with cerulenin treatment in bNHL cells or with FASN siRNA in SUDHL10 cells. Western blot represents the extent of siRNA-mediated FASN knockdown observed in SUDHL10 cells. Bar graph represents the ratio of FASN protein expression normalized by total β-actin for equal loading. Absolute concentrations corresponding to these fold changes are included in **Supplementary Table S1**. **(E)** Flow cytometry of ClickIT 5-EU pulse-labeled SUDHL10 cells show decreased fluorescence intensity indicative of RNA global transcriptional activity occurring with cerulenin treatment in SUDHL10 compared to control. **(F, G)** Bar graph representing changes in the concentration of dNTPs following cerulenin treatment in SUDHL10 cells. Confocal imaging of ClickIT 5-EdU pulse-labeled SUDHL10 cells show decreased 5-EdU (red) incorporation in DNA against DAPI (blue) or merged occurring with cerulenin treatment in SUDHL10 compared to control. All experiments were performed in triplicates; the error bars in all bar graphs represent the standard deviations of mean, and the significant difference between control and treatment is indicated by an asterisk (**p < 0.05 and ***p < 0.005) statistically determined by Student’s *t*-test. **(H)** Histogram representing changes in cell cycle occurring with cerulenin treatment based on the flow cytometry of propidium-stained SUDHL10 cells at 48 h **(H)** Overlaid histogram representing cell cycle changes comparing cerulenin with control at 48 h in SUDHL10 cells, detected by propidium iodide staining and flow cytometry analysis.

Furthermore, the VIP score-based ranking identified 6PG as second among the top 15 metabolites impacted by FASN inhibition (**Figure 2B**). Utilizing “PatternHunter” (Metaboanalyst package), a negative correlation (<-0.5) (light blue bars) between 6PG (light pink bar) with R5P and metabolites from *de novo* purine/pyrimidine biosynthesis (inosine monophosphate-IMP, AICAR, AMP, GMP, UMP, alanine, and aspartate) were observed in cerulenin-treated SUDHL10 cells (**Figure 3D**). Considering that R5P is a PGDH end-product and a precursor located upstream prior to *de novo* nucleotide biosynthesis, our results suggest that PGDH perturbation and the reduction in nucleotide levels are both relevant to FASN inhibition, as observed in SUDHL10 cells. We then investigated the impact of FASN inhibition on *de novo* nucleotide biosynthesis by the following experiments: pulse labeling and isotope tracing of carbon flow from C^13^-glucose into nucleotides showed a reduction in the proportion of C^13^-labeled C5 fractions associated with nucleotide monophosphates (AMP, CMP, GMP, and IMP) while increasing the amounts of 6PG-labeled fractions, confirming that PGDH and nucleotide biosynthesis are suppressed by FASN inhibition with cerulenin (**Figures 4A, B**). Further confirmatory quantitative mass spectrometry assessments using multiple bNHL cell lines (SUDHL10, SUDHL4, and Raji) treated with cerulenin or silencing FASN by siRNA in SUDHL10 cells also revealed that nucleotide depletion is a consistent feature in FASN inhibition, shown as a bar graph by percent changes (**Figures 4C, D**) and as absolute concentrations presented in **Supplementary Table S2**.

With the decreased nucleotide biosynthesis, we next examined the effects of FASN inhibition on nucleic acid metabolism (DNA/RNA synthesis) and cell cycle. 5-Ethynyl uridine (EU) incorporation and tracking nascent RNA synthesis revealed a net reduction in global transcriptional activity occurring with cerulenin treatment, detected as decreased EU RNA incorporation by flow cytometry (**Figure 4E**). Deoxyribonucleotides (dNTPs) synthesized from ribonucleotides are utilized in DNA replication; therefore, we first investigated the effect of FASN inhibition on dNTP synthesis. The results from the dNTP evaluation, represented as bar graphs, indicate a significant reduction in the absolute levels of dNTPs (dATP, dCTP, dTTP, and dGTP) occurring with cerulenin treatment in SUDHL10 cells (**Figure 4F**). Subsequently, the 5-ethynyl deoxy-uridine (EdU) incorporation assay showed a marked reduction in EdU labeling of DNA (by confocal microscopy), demonstrating impaired DNA synthesis occurring in the presence of cerulenin treatment in SUDHL10 cells (**Figure 4G**). Finally, with decreased DNA synthesis, we also observed a reduction in S phase population and increased accumulation of cells in G1, impacting the cell cycle as observed from PI staining and flow cytometry performed with cerulenin-treated SUDHL10 cells (**Figure 4H**).

### Enzymatic Coupling of FASN–PGDH Activities

**Figure 5:**
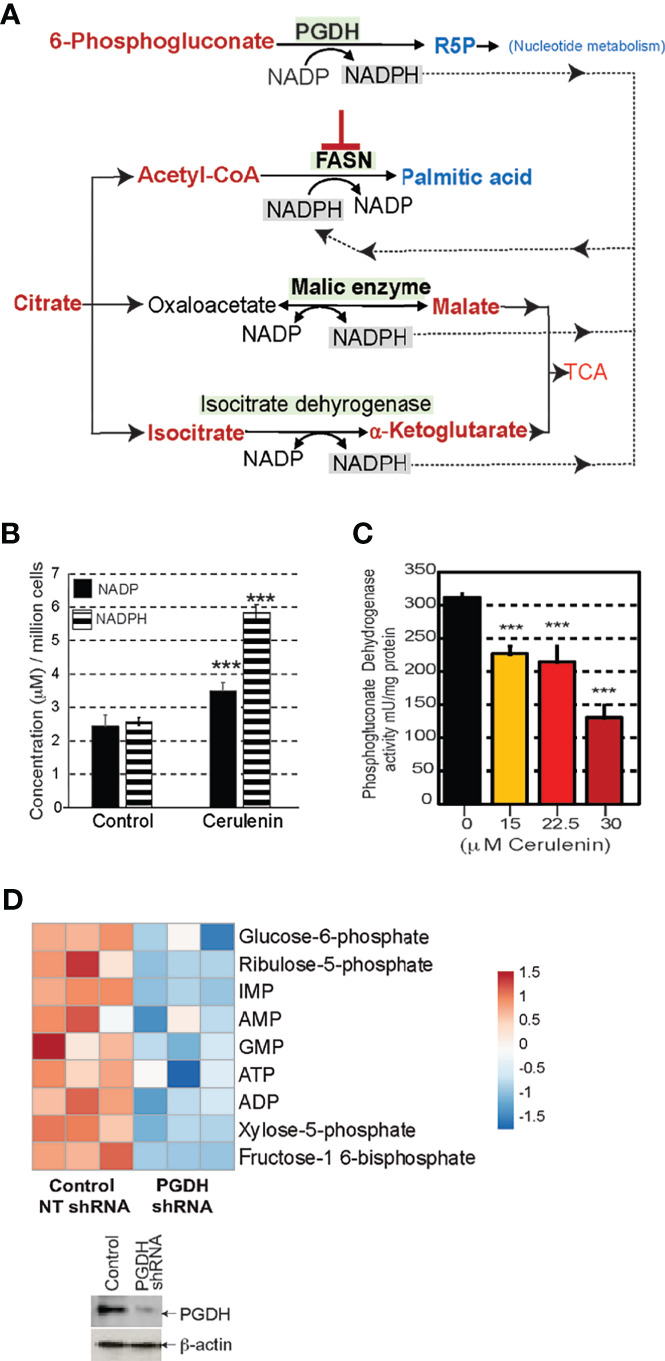
FASN inhibition interrupts phosphogluconate dehydrogenase (PGDH) activity. **(A)** Schematic representation of the metabolic impacts of FASN inhibition on potential enzymatic sources of NADPH for FASN activity; elevated metabolites are shown in red, and decreased metabolites are shown in blue. **(B)** Bar graph representing changes in concentrations of NADP or NADPH comparing untreated control and cerulenin treatment (on x-axis), and concentration represented as μM/million cells (on y-axis) in SUDHL10 cells. Data represented are based on averages from experimental triplicates, comparing cerulenin-treated cells with untreated control, with *** denoting *P <*0.001. **(C)** Bar graph representing PGDH enzymatic activity in SUDHL10 cells treated with cerulenin, with the concentration shown on x-axis, and enzyme activity normalized by mU/mg of total protein (on y-axis). All experiments were performed in triplicates. The error bars in all bar graphs represent the standard deviations of mean, and the significant difference between control and treatment is indicated by an asterisk (***p < 0.005), statistically determined by Student’s *t*-test. **(D)** Heat map representing the metabolic impacts of shRNA-mediated PGDH knockdown compared with non-targeted control in SUDHL4 cells, shown as log_2_-transformed, row-centered data from experimental triplicates. Western blot representing the extent of PGDH knockdown observed with shRNA-mediated RNA silencing in SUDHL4 cells.

FASN inhibition resulted in nucleotide depletion and impacted DNA/RNA metabolism and the cell cycle. It is likely that the perturbation of PGDH could be relevant for the negative impacts observed with nucleotide metabolism associated with FASN inhibition. We, therefore, focused our investigations towards delineating the mechanistic link between FASN inhibition and the perturbation of PGDH activity. Sources of cytosolic NADPH for FASN activity include PGDH, malic enzyme, and isocitrate dehydrogenase; however, with FASN inhibition, the metabolic profiles show that only PGDH activity was impacted (shown in blue), while the other enzymes remained unaffected (**Figure 5A**). Moreover, PGDH is the only rate-limiting unidirectional enzyme susceptible to allosteric inhibition by NADPH. From these evidence, we concluded that PGDH is the only NADPH-yielding enzyme that was responsive to FASN inhibition. The results from NADP/NADPH quantification by NADP/NADPH-Glo assay showed a significant fold increase in both NADP and NADPH levels (*P* < 0.001) occurring with cerulenin treatment in SUDH10 cells (**Figure 5B**). While NADPH accumulation is expected with FASN inhibition, the reason for the observed NADP increase seems unclear. To better understand these metabolic impacts, we first performed enzyme activity assays, using SUDHL10 cells, and observed a decrease in PGDH activity occurring in a cerulenin-concentration-dependent manner (**Figure 5C**). Our western blot analysis revealed a stable PGDH protein expression (**Supplementary Figure S4**), indicating that PGDH is metabolically repressed under FASN inhibition. Similar inhibitions of PGDH enzyme activity by cerulenin were observed in other NHL cells (SUDHL4 and Raji) (data not shown). Notably, the expression of PPP enzyme G6PDH and dependent antioxidant enzymes showed a dynamic increase in protein expression with FASN inhibition, indicating that PGDH is the only unregulated expression function in this pathway (**Supplementary Figures S4B–E**). Since PGDH is the rate-limiting bridge between PPP and nucleotide biosynthesis, we investigated the impact of PGDH silencing using shRNA. The results from this experiment showed that ribulose-5-phosphate (PGDH product) and inosine monophosphate (precursor associated with *de novo* nucleotide biosynthesis) were reduced (**Figure 5D**), with decreased PGDH. Therefore, it is now apparent that PGDH perturbation by both FASN inhibition and RNAi results in negatively impacting the nucleotide metabolism.

**Figure 6:**
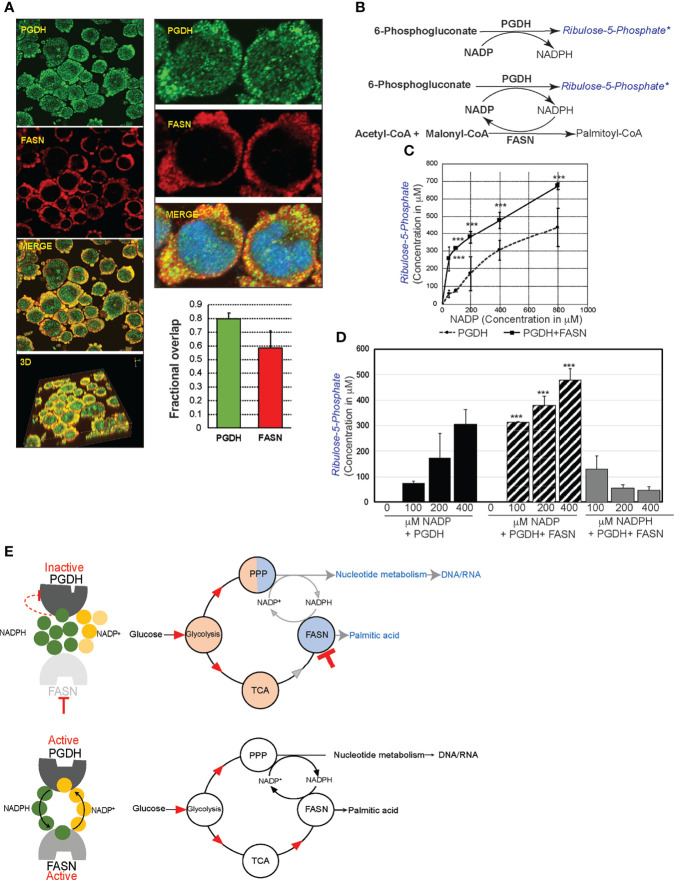
FASN and phosphogluconate dehydrogenase (PGDH) are metabolically synergistic enzymes. **(A)** Confocal microscopy of SUDHL10 cells stained with rabbit anti-human PGDH (green) and mouse anti-human FASN antibody (red) as primary antibodies and donkey anti-rabbit AlexaFluor-488 and goat anti-mouse AlexaFluor-594 as secondary antibodies. Images were acquired using ×40 objectives and ×2.5 optical zoom, shown as individual channels or merged or 3D-rendered volume. Bar graph representing colocalization as overlap by correlation between PGDH and FASN expression, with error bars representing averages from an analysis of three independent images by ImageJ. **(B)** Schematic representation of cell-free assay experimental design. Shown in black are the reactants supplied in the reaction buffer, and shown in blue are the products. FASN–PGDH double-enzyme reactions were performed with either NADP or NADPH as cofactors. “*” refers to reaction product quantified by mass spectrometry. **(C)** Line graph representing ribulose-5-phosphate quantification (y-axis) by mass spectrometry from PGDH-catalyzed reaction consisting of increasing concentrations of NADP (x-axis). **(D)** Bar graph representing the quantification of ribulose-5-phosphate (y-axis) and comparison of increasing concentrations of NADP-driven PGDH or PGDH+FASN-catalyzed reaction and NADPH-driven PGDH+FASN-catalyzed reactions. The error bars represent the standard deviations of mean and the significant differences between each concentration of NADP-driven PGDH reaction, with NADP- or NADPH-driven PGDH+FASN-catalyzed reaction indicated by an asterisk (***p < 0.001). **(E)** Diagram representing the allosteric stimulation or inhibitory effects of NADP^+^ and NADPH on PGDH and summarizing the impact of FASN inhibition on overall metabolic pools (shaded in red indicating increases or blue indicating decreases) in comparison with metabolic flow in uninhibited cells.

Mechanistically, perturbation of PGDH activity is feasible through allosteric feedback inhibition caused by NADPH accumulation resulting from FASN inhibition (**Figure 5B**). PGDH is sensitive to allosteric inhibition by NADPH (Ki value, 0.03 μM) (source: BRENDA, www.brenda-enzymes.org). Thus, for NADPH accumulation by FASN inhibition to impact PGDH function, both of these enzymes must be localized in close proximity. The results from colocalization studies based on immunofluorescence staining and confocal imaging analysis of SUDHL10 cells showed that both PGDH (green) and FASN (red) were localized in the cytosol, with few punctate nuclear distributions observed with PGDH (**Figure 6A**). A colocalization analysis by Manders coefficient method (JaCoP, ImageJ plugin) performed using three independent sets of images determined that 0.79 ± 0.04 fraction of PGDH overlapped with FASN, and 0.58 ± 0.12 fraction of FASN likewise overlapped with PGDH in SUDHL10 cells (**Figure 6A**). Similar results with overlaps and colocalization patterns for FASN and PGDH were also observed from staining with independent sets of antibodies (FASN #sc-398977 and PGD #sc48357, Santa Cruz Biotechnology, Dallas, TX) and additional SUDHL4 cell line (data not shown).

The FASN and PGDH colocalization patterns indicate that both of these enzymes are proximally localized, and, therefore, shuttling of NADP–NADPH between these enzymes could occur within the cells. In order to determine whether reciprocal shuttling of NADP–NADPH occurs and influences FASN and PGDH, we performed the following cell-free enzyme assays: first, PGDH catalytic activity was quantified in the presence of increasing concentrations of cofactor, NADP, and fixed substrate concentration (6PG). The results from mass spectrometry indicate that the stimulation of PGDH activity occurred in the presence of an increased NADP concentration (50–800 μM), resulting in an increase of ribulose-5-phosphate (R5P) synthesis (57 ± 20–437 ± 111 μM). Next, we observed that coupling FASN reaction with NADP-driven PGHD resulted in a significantly increased R5P synthesis at 256 ± 68μM (*P* < 0.001), compared to R5P synthesis 57 ± 20μM by PGDH alone in the reactions performed using 50 μM NADP (**Figures 6B, D**). Moreover, we observed that FASN presence led to decreasing the Km for NADP from 22.1 to 11.5 μM and the acceleration of PGDH reaction velocity, demonstrating that FASN is exerting a positive influence on PGDH activity. The spectrophotometric monitoring also showed a reduction in the kinetics of NADPH appearance in the presence of FASN (compared to PGDH without FASN) (**Supplementary Figure S4F**). These results together indicate that PGDH activity is stimulated by NADP and that the removal of NADPH in the presence of FASN leads to an accelerated synthesis of R5P by PGDH. Similarly, in FASN–PGDH coupled reactions consisting NADPH, an incremental presence of NADPH resulted in the suppression of R5P synthesis, indicating that NADPH exerts an inhibitory effect on PGDH activity (**Supplementary Figures 6B, D**). In conclusion, FASN enhances PGDH activity *via* the regeneration of allosteric stimulator NADP and reducing the levels of the allosteric inhibitor NADPH (**Figure 6D**). The results observed from these experiments suggest that NADPH accumulation could be biologically responsible for PGDH inactivation in the presence of FASN inhibition (**Figure 5C**). Thus, FASN inhibition and perturbation of PGDH could lead to the accumulation of glycolytic and citric acid cycle intermediates and contributed to nucleotide depletion as observed and summarized in **Figures 3B** and **6E**.

### Onco-Metabolic Implications of Glucose, Nucleotide, and Lipid Metabolism in Lymphoma

**Figure 7:**
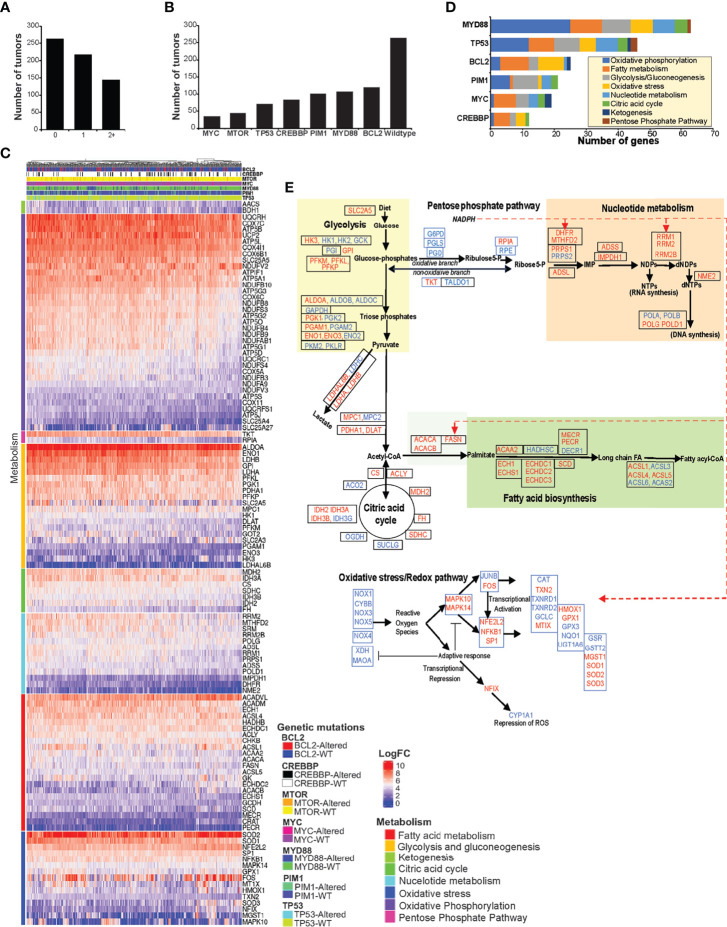
DLBCL metabolic transcriptome. **(A, B)** Bar graphs represent the proportion and distribution by number of tumors, consisting of wild-type, single, or multiple mutations in MYC, MTOR, TP53, CREBBP, PIM1, MYD88, and TP53. **(C)** Heat map representing the differential expression of metabolic genes, significant by single-gene mutation with *P <*0.005, comparing tumors that consist of wild-type or mutant MYC, MTOR, TP53, CREBBP, PIM1, MYD88, and TP53 genes (*n* = 624). **(D)** Bar graph representing the number of genes overexpressed by metabolic pathways and distribution by genetic mutations. **(E)** Flow chart representing the mapping of differentially expressed genes corresponding to glucose, pentose phosphate pathway, lipid, and redox metabolic pathways. The upregulated genes are indicated in red color, and the unaffected genes are indicated in blue color. Dashed red lines indicate NADPH-related enzymes.

The results from *in vitro* metabolic and enzymatic assessments showed FASN and PPP as interrelated metabolic functions linked with nucleotide metabolism. Ample nucleotide supply is necessary for proliferative functions, including for the support of malignant cell proliferation. Therefore, altering nucleotide metabolism is an end-point and major end-point for regulation by oncogenic factors. In this context, we analyzed RNA seq data from *n* = 624 NHL tumors consisting of genetic mutations (*n* = 361) with unaltered (*n* = 263) TP53, MYC, BCL2, mTOR, MYD88, PIM2, and CREBP and identified differentially expressed metabolic genes (**Figure 7A**). Tumors with mutations (*n* = 361) included 144 tumors with two or more multiple mutations (**Figure 7B**). A total of 116/241 genes were altogether identified as differentially expressed metabolic genes based on significant cutoff (*P* < 0.005) in tumors consisting of mutations in TP53, MYC, BCL2, mTOR, MYD88, PIM2, and CREBP against tumors that are wild type for these genes. The differentially expressed genes represented in the heat map (**Figure 7C**) indicate elevated genes (log_2_ two- to sevenfold) associated with fatty acid, glycolysis, gluconeogenesis, citric acid cycle, ketogenesis, PPP, nucleotide, oxidative phosphorylation, and oxidative stress metabolism (**Figure 7C**).

Alterations in MYD88 and TP53 resulted in the most numbers of metabolic genes being upregulated, 64 and 46, respectively; however, other genetic mutations also yielded similar distributions in the upregulated metabolic gene expression patterns (**Figure 7D**). Furthermore, of 361 tumors with genetic mutations, 144 tumors carried two or more genetic mutations, indicating that the upregulation of metabolic gene expression could be under the influence of multiple oncogenic mutations in these NHL tumors. Further mapping of elevated genes (red) to corresponding metabolic pathways indicate that >95% of metabolic steps associated with glucose, PPP, lipid, and nucleotide metabolism were increased (**Figure 7D**), but regardless of mutational origins. Interestingly, genes related to oxidative PPP (G6PD, PGLS, and PGDH) were not elevated, but genes relevant to metabolic feeder pathways (glucose and lipid metabolism) and dependent processes (nucleotide metabolism) were found to be elevated in these tumors. These gene expression patterns altogether suggest that the enzymatic modulation of oxidative PPP activity *via* elevated FASN activity could be necessary for influencing PPP and nucleotide biosynthesis in these NHL tumors.

## Discussion

Intracellular nucleotide concentrations fluctuate continuously during cell progression, dictating the overall fate, frequencies, and timings of cell division (32–34). While nucleotide biosynthesis and proliferation are tightly coordinated processes in normal cells, a disproportional increase in nucleotide levels is commonly observed in malignant cells (35). Most importantly, the lack of carrier protein and the presence of negative charges are barriers that require endogenous nucleotide biosynthesis as a metabolic essentiality for sustaining cellular proliferation (36). While *de novo* biosynthesis primarily caters the nucleotide supply for DNA replication, salvage biosynthesis often supports only low-demand functions, such as DNA repair, *etc*. (36). Thus, promotion of *de novo* nucleotide biosynthesis is the landmark and end-point for transcriptional upregulation by various oncogenic factors, including by MYC, RAS, PI3K, AKT, Rb, mTOR, MAPK, NFκB, *etc*. (35, 37). Increased nucleotide metabolism is absolutely necessary for gaining proliferative advantage in malignancy; therefore, malignant cells generally maintain excess (approximately three- to sixfold higher) amounts of nucleotides compared to normal counterparts (38). The correlation between elevated nucleotide metabolism and poor clinical outcome is reported in both hematological and solid tumors (35, 38). Our metabolic and transcriptomic assessments with FASN inhibition demonstrate that NADPH utilization and NADP regeneration by FASN could allow PGDH to remain active and sustain nucleotide biosynthesis. Furthermore, the increased expression of genes relevant to FASN metabolism, glycolysis, citric acid cycle, and nucleotide metabolism, but not of genes associated with oxidative PPP, underscores the presence of FASN as enzymatic necessity for promoting nucleotide metabolism in the tumors.

Lipids are physiologically abundant molecules (free fatty acids, triglycerides, VLDL, LDL, and HDL), and malignant cells are known to utilize and integrate exogenous lipids with greater preference (39). Furthermore, FASN-mediated lipogenesis is an energetically expensive reaction that consumes massive amounts of energy (through seven ATP, 14 NADPH, and eight acetyl-CoA used per molecule of palmitate synthesis) (40). Therefore, *de novo* lipogenesis is a biologically unfavorable metabolic reaction, an energy competitor which is unbeneficial to malignant cell proliferation. However, in the context of extra-lipogenic function, it has been shown that FASN activity could serve as a positive influencer and metabolic driver of glycolysis and citric acid cycle under hypoxia (9). Similarly, rapid bursts of FASN activity accompanied with increased glucose uptake and metabolism are observed during the proliferative expansion of B and T lymphocytes (41). While in lipogenic tissues *de novo* lipogenesis could be the primary function of FASN, extra-lipogenic role could be a significant metabolic function of FASN for non-lipogenic tissues.

The results from our experiments demonstrate that extra-lipogenic activity could involve leveraging FASN substrate and co-factor dependency for promoting interlinked metabolic activities. NADP is an allosteric stimulator of PGDH and inhibitor of FASN. Similarly, NADPH is an allosteric stimulator of FASN and inhibitor of PGDH. Thus, NADP and NADPH turnover is reciprocally beneficial for sustaining FASN and PGDH metabolic activity. Therefore, mitogenic stimulation, triggering *de novo* lipogenic activity as observed in B lymphocytes (41, 42), could be pertinent to diverting glucose carbon *via* PPP and nucleotide biosynthesis for supporting cell proliferation. PPP and nucleotide metabolism is dynamically influenced by the physiological demands. In resting cells, R5P synthesized from PPP is shunted back to glycolysis (43); during oxidative stress and DNA damage, G6PDH-coupled glutathione reaction caters to the supply of nucleotides for DNA repair and NADPH for antioxidant defense (44). G6PDH and PGDH are both rate-limiting PPP metabolic steps subject to allosteric inhibition by NADPH (Ki for PGDH is ~0.03 and for G6PDH is ~0.017 μM; source: average Ki values for human enzymes; BRENDA, www.brenda-enzymes.org). Interestingly, PGDH exhibits lower Ki for NADPH compared to G6PDH; thus, PGDH becomes more vulnerable to inhibition by NADPH. Similarly, FASN has remarkably low Km for NADPH compared to PPP-dependent antioxidant enzymes (~Km values for NADPH: FASN—0.005 μM, GR—0.008 μM, TR—0.088 μM, NQO1—0.24 μM; source: BRENDA, www.brenda-enzymes.org). Thus, FASN with low Km for NADPH and PGDH with low Ki NADP could function as ideal enzymatic partners for NADP–NADPH recycling activity. Considering that FASN-inhibition-associated NADPH accumulation inhibits PGDH activity, we conclude that FASN, *via* a NADP–NADPH recycling process, could lead to sustained PGDH activity, as summarized in models shown in **Figure 6E**.

PPP is considered as a central integrator of glucose, nucleotide, lipid, and oxidative stress metabolism (37, 43). Overwhelming evidence indicates that nucleotide metabolism is the end-point for oncogenic functions mediated by MYC, PI3K, NFκB, AP1, c-jun, c-fos, GATA1, FOXO, HOX, E2F, and STAT1 (35, 45). The results from RNA seq data analysis comparing the impact of genetic mutations of TP53, MYD88, MYC, BCL2, PIM1, and CREBBP show that metabolic genes included in nucleotide biosynthesis are upregulated in NHL tumors (**Figure 7**). With FASN upregulation and unapparent effect on oxidative PPP genes, FASN is likely to function as an enzymatic driver of oxidative PPP in lymphoma tumors.

## Conclusion

In summary, FASN upregulation, generally ascribed with lipogenic function in malignancy, appears to be necessary for promoting nucleotides in lymphoma. The results from FASN inhibition with cerulenin treatment show the transcriptomic downregulation of nucleotide metabolism and cell cycle processes (**Supplementary Figure S2**) to be correlated with reduced nucleotide biosynthesis, nucleic acid metabolism, and cell cycle impairment (**Figure 4**). Furthermore, impairment of nucleotide biosynthesis caused by NADPH accumulation and interruption of PGDH activity by FASN inhibition resulted in the metabolic accumulations in PPP, while citrate accumulation resulted in the metabolic accumulations within the citric acid cycle and glycolysis (**Figure 3B**). Based on these observations, we conclude that FASN, *via* NADPH and citrate utilization in palmitic acid biosynthesis, plays a central role in the integration of glucose metabolism with nucleotide biosynthesis. Thus, the results from our experiments and prior reports showing that tumor cells exhibit preferential utilization for extracellular lipids (39) suggest that extralipogenic function should be the primary metabolic dysfunction of FASN in cancer.

The analysis of the NHL transcriptome from *n* = 624 lymphoma patients showing upregulations in the expression of FASN nucleotide metabolic genes, but not with those of oxidative PPP in the presence of lymphomagenic mutations (**Figure 7**), underpins the necessity of FASN to function as a metabolic driver of nucleotide synthesis in cancer. Although targeting FASN impairs the cell cycle (**Figure 4H**), the impact on decreasing nucleotide levels (**Figure 4C**) provides unique opportunities for combining FASN inhibitory drugs with antinucleoside analogs—for example, we observed that combining 5FU with FASN inhibitor in fact resulted in increased apoptotic cell death (**Supplementary Figure S4G**), possibly *via* the robust incorporation of 5FU into the nucleic acid structures. Finally, experiments based on cell-free enzyme assay demonstrating that NADP/NADPH recycling occurs between FASN and PGDH and the resultant increase in the R5P synthesis implicate that PPP is, mechanistically, a FASN-dependent metabolic function (**Figure 6**).

We altogether conclude that FASN PGDH enzymes exhibit metabolic cooperativity and facilitate the flow of glucose carbons through PPP metabolism into the nucleotide biosynthesis in lymphoma.

## Data Availability Statement

The datasets presented in this study can be found in online repositories. The names of the repository/repositories and accession number(s) can be found in the article/**Supplementary Material**.

## Ethics Statement

The results from the RNA seq of NHL patients were obtained from public datasets previously published and authored by SD. For original investigation, anonymized lymphoma specimens were processed in accordance with protocol approved by the Institutional Review Board at Duke University.

## Author Contributions

DR conceptualized the study, conducted experiments, and performed overall research, data analysis, and manuscript writing. AB performed transcriptomic data analysis and manuscript writing. Research interns NA, FP, and PP conducted cell viability assays. MM performed flow cytometry assays. FL and WK provided funding support and performed data analysis and manuscript writing NM conducted lipid extraction and analysis, and GD provided funding support and performed mass spectrometry lipid assessment, data analysis and manuscript writing. XS performed mass spectrometry profiling of polar metabolites. RV performed cell-free enzyme assay, and MR provided recombinant FASN and performed the analysis of cell-free enzyme assay data and manuscript review. SD shared RNA seq data, and YC performed bioinformatic analysis. AE provided funding support, designed the research, and performed data analysis and manuscript writing. All authors contributed to the article and approved the submitted version.

## Funding

FL, WK, and AE were supported by Our Danny cancer fund. DR, GD, and AE were supported by Tufts Medical Center–Human Nutrition Research Center on Aging pilot fund. NM and GD were supported by the US Department of Agriculture (agreement no. 58-1950-4-401). DR and AE were supported by 3-V Biosciences. The research service of XS was supported by the Metabolomics shared resource of Rutgers Cancer Institute of New Jersey (P30CA072720). BK was supported by NIH/NIAID R01 AI150451 and AI136581. The research service of YC was generated by Biomedical Informatics shared resource of Rutgers Cancer Institute of New Jersey, supported, in part, with funding from NCI-CCSG P30CA072720-5917. 3-V Biosciences was not involved in the study design, collection, analysis, interpretation of data, the writing of this article, or the decision to submit it for publication.

## Author Disclaimer

Any opinions, findings, conclusions or recommendations expressed in this publication are those of the authors and do not necessarily reflect the view of the U S Department of Agriculture.

## Conflict of Interest

AE: Advisory board (with honorarium): Bayer, Seattle Genetics, Affimed, Verastem, Pharmacyclics, Research to Practice, and Physician Education Resource. Research support: Takeda, Seattle Genetics, Merck, NIH/ NCI, Leukemia and Lymphoma Society, and ORIEN.

The remaining authors declare that the research was conducted in the absence of any commercial or financial relationships that could be construed as a potential conflict of interest.

## Publisher’s Note

All claims expressed in this article are solely those of the authors and do not necessarily represent those of their affiliated organizations, or those of the publisher, the editors and the reviewers. Any product that may be evaluated in this article, or claim that may be made by its manufacturer, is not guaranteed or endorsed by the publisher.

## Supplementary Material

Supplementary Figure S1 Identification of differentially expressed genes by transcriptomic profiling with FASN inhibitors in bNHL cells. Hierarchical clustering of genes and Euclidean distance calculation for 48 hours of 12.5µM cerulenin treatment on **(A)** SUDHL2 cells with One-Way ANOVA, FDR < 0.1 (141 genes), **(B)** SUDHL10 cells with LIMMA analysis, FDR < 0.05 (1600 genes) **(C)** SUDHL4 cells with One-Way ANOVA, FDR < 0.05 (242 genes), **(D)** Raji cells with LIMMA analysis, FDR < 0.05 (1092 genes). **(E)** Hierarchical clustering of genes and Euclidean distance calculation for 48 hours of TVB3166 or 3657 in SUDHL2, SUDHL10 and Raji cells with One-Way ANOVA, FDR < 0.05 (8872 genes). **(F)** Principal component analysis plot of TVB3166 or TVB3567 treated bNHL cells show global differences between the experimental conditions and the untreated controls for SUDHL2, SUDHL10 and Raji cells. **(G)** Scatter plot represents fold changes in the gene expression from the transcriptome of TVB3166 or 3657 or cerlulenin treated bNHL cells (no significant difference were detected in the gene expression pattern with cerulenin treatment performed in the presence of 1% or 10% serum containing medium, data not shown), with whiskers showing the range of the outliers, with max and min values as O and the 1 and 99th percentile outliers as X. Individual data points are shown on the left of box plots as filled circles. Dotted red lines show the 1.2 log2 fold-change cutoff.

Supplementary Figure S2–S3 GSEA analysis of global biological responses to FASN inhibition. Network representation of Gene Set Enrichment Analysis (GSEA) for Reactome gene sets for **(A)** cerulenin or **(B)** and **S3** for TVB3166 or TVB3567 treatment, 48 hours versus untreated control bNHL cells, SUDHL2, SUDHL4, SUDHL10 and Raji cells. Leading edge analysis with a FDR<0.05 determined significant gene sets enriched for each group. The size of each node reflects the amounts of genes involved for each gene set. The edge thickness (green lines) represents the number of genes associated with the overlap of two gene sets (or nodes) that the edge connects. Clusters in each grouping were named according to common functions. Upregulated gene sets denoted with red color and downregulated gene sets were denoted by blue color.

Supplementary Figure S4 Metabolic and key gene responses to cerulenin treatment in bNHL cells. **(A)** Line graphs represents spectrophotometric detection of NADPH appearance (y-axis) as rate of change in OD (optical density at 340nM wavelength from cell-free enzyme assays consisting increasing concentrations of NADP (x-axis) comparing PGDH alone or PGDH+FASN catalyzed reactions. **(B)** Lineweaver-Burk plot represents PGDH reaction velocity in the presence of increasing NADP concentration, and the effect of coupling this reaction with FASN enzymatic activity. **(C)** Key significant genes and network analysis identified from transcriptomic analysis of cerulenin treated with SUDHL10 cells reveal interactions occurring between G6PDH (NADPH generating PPP, rate-limiting enzyme from first step) and NADPH dependent enzymes GSR, TXNRD1 and NQO1 with FASN inhibition. **(D)** Schematic representation of pentose phosphate pathway steps associated with NADPH generation. **(E)** Bar graphs represents log fold change (y-axis) in G6PDH, GSR, TXNRD1, NQO1 genes expression induced by cerulenin treatment in multiple bNHL cells (x-axis), **(F)** Western blot analysis show changes in protein levels associated with key gene responses to cerulenin treatment in NHL cells. **(G)** Annexin-V staining for apoptosis and analysis by flow cytometry show that cerulenin and 5FU combination results in increased cell death compared to single agent treatments in SUDHL10 cells at 96 hours.

Supplementary Table S1 Pathway enrichment by multi-omic analysis comparing cerulenin transcriptome and metabolome. List of pathways determined using significant genes and metabolites from cerulenin treated SUDHL10 cells analyzed based on pathway centrality and degree of enrichment for determination of high impact pathways shown in Figure 2E, is included in this table. Impact scores, FDR, P values, number of molecules and hits are included in this table.

Supplementary Table S2 Concentration of nucleotide pools in cerulenin treated bNHL cells. Quantitative mass spectrometry analysis of cerulenin treated bNHL cells, normalized per million cells, represented in μM or *ion counts.

## References

[1] Yoshii Y Furukawa T Saga T Fujibayashi Y. Acetate/acetyl-CoA Metabolism Associated With Cancer Fatty Acid Synthesis: Overview and Application. Cancer Lett (2015) 356:211–6. doi: 10.1016/j.canlet.2014.02.019 24569091

[2] Mashima T Seimiya H Tsuruo T. *De Novo* Fatty-Acid Synthesis and Related Pathways as Molecular Targets for Cancer Therapy. Br J Cancer (2009) 100:1369–72. doi: 10.1038/sj.bjc.6605007 PMC2694429 19352381

[3] Rodwell V Bender D Botham K Kennelly P Weil P. Harper’s Illustrated Biochemistry. (2018).

[4] Frey RS Gao X Javaid K Siddiqui SS Rahman A Malik AB. Phosphatidylinositol 3-Kinase Gamma Signaling Through Protein Kinase Czeta Induces NADPH Oxidase-Mediated Oxidant Generation and NF-kappaB Activation in Endothelial Cells. J Biol Chem (2006) 281:16128–38. doi: 10.1074/jbc.M508810200 16527821

[5] Kuhajda FP. Fatty Acid Synthase and Cancer: New Application of an Old Pathway. Cancer Res (2006) 66:5977–80. doi: 10.1158/0008-5472.CAN-05-4673 16778164

[6] Flavin R Peluso S Nguyen PL Loda M. Fatty Acid Synthase as a Potential Therapeutic Target in Cancer. Future Oncol (2010) 6:551–62. doi: 10.2217/fon.10.11 PMC3197858 20373869

[7] Flaumenhaft R Sim DS. Protein Palmitoylation in Signal Transduction of Hematopoietic Cells. Hematology (2005) 10:511–9. doi: 10.1080/10245330500141507 16321817

[8] Fragoso R Ren D Zhang X Su MW Burakoff SJ Jin YJ. Lipid Raft Distribution of CD4 Depends on its Palmitoylation and Association With Lck, and Evidence for CD4-Induced Lipid Raft Aggregation as an Additional Mechanism to Enhance CD3 Signaling. J Immunol (2003) 170:913–21. doi: 10.4049/jimmunol.170.2.913 12517957

[9] Menendez JA Lupu R. Fatty Acid Synthase and the Lipogenic Phenotype in Cancer Pathogenesis. Nat Rev Cancer (2007) 7:763–77. doi: 10.1038/nrc2222 17882277

[10] Zhang J Grubor V Love CL Banerjee A Richards KL Mieczkowski PA. Genetic Heterogeneity of Diffuse Large B-Cell Lymphoma. Proc Natl Acad Sci USA (2013) 110:1398–403. doi: 10.1073/pnas.1205299110 PMC3557051 23292937

[11] Danilova OV Dumont LJ Levy NB Lansigan F Kinlaw WB Danilov AV. FASN and CD36 Predict Survival in Rituximab-Treated Diffuse Large B-Cell Lymphoma. J Hematop (2013) 6:11–8. doi: 10.1007/s12308-012-0166-4 PMC4415532 25937841

[12] Nowakowski GS Czuczman MS. ABC, GCB, and Double-Hit Diffuse Large B-Cell Lymphoma: Does Subtype Make a Difference in Therapy Selection? Am Soc Clin Oncol Educ Book (2015), e449–57. doi: 10.14694/EdBook_AM.2015.35.e449 25993209

[13] Baudino TA. Targeted Cancer Therapy: The Next Generation of Cancer Treatment. Curr Drug Discovery Technol (2015) 12:3–20. doi: 10.2174/1570163812666150602144310 26033233

[14] Funabashi H Kawaguchi A Tomoda H Omura S Okuda S Iwasaki S. Binding Site of Cerulenin in Fatty Acid Synthetase. J Biochem (1989) 105:751–5. doi: 10.1093/oxfordjournals.jbchem.a122739 2666407

[15] Ventura R Mordec K Waszczuk J Wang Z Lai J Fridlib M. Inhibition of *De Novo* Palmitate Synthesis by Fatty Acid Synthase Induces Apoptosis in Tumor Cells by Remodeling Cell Membranes, Inhibiting Signaling Pathways, and Reprogramming Gene Expression. EBioMedicine (2015) 2:808–24. doi: 10.1016/j.ebiom.2015.06.020 PMC4563160 26425687

[16] Heuer TS Ventura R Mordec K Lai J Fridlib M Buckley D. FASN Inhibition and Taxane Treatment Combine to Enhance Anti-Tumor Efficacy in Diverse Xenograft Tumor Models Through Disruption of Tubulin Palmitoylation and Microtubule Organization and FASN Inhibition-Mediated Effects on Oncogenic Signaling and Gene Expression. EBioMedicine (2017) 16:51–62. doi: 10.1016/j.ebiom.2016.12.012 28159572 PMC5474427

[17] Ravi D Beheshti A Abermil N Passero F Sharma J Coyle M. Proteasomal Inhibition by Ixazomib Induces CHK1 and MYC-Dependent Cell Death in T-Cell and Hodgkin Lymphoma. Cancer Res (2016) 76:3319–31. doi: 10.1158/0008-5472.CAN-15-2477 PMC5315412 26988986

[18] Beheshti A Neuberg D McDonald JT Vanderburg CR Evens AM. The Impact of Age and Sex in DLBCL: Systems Biology Analyses Identify Distinct Molecular Changes and Signaling Networks. Cancer Inform (2015) 14:141–8. doi: 10.4137/CIN.S34144 PMC4676434 26691437

[19] Beheshti A Wage J McDonald JT Lamont C Peluso M Hahnfeldt P. Tumor-Host Signaling Interaction Reveals a Systemic, Age-Dependent Splenic Immune Influence on Tumor Development. Oncotarget (2015) 6:35419–32. doi: 10.18632/oncotarget.6214 PMC4742115 26497558

[20] Reddy A Zhang J Davis NS Moffitt AB Love CL Waldrop A. Genetic and Functional Drivers of Diffuse Large B Cell Lymphoma. Cell (2017) 171:481–94 e15. doi: 10.1016/j.cell.2017.09.027 28985567 PMC5659841

[21] Gu Z Eils R Schlesner M. Complex Heatmaps Reveal Patterns and Correlations in Multidimensional Genomic Data. Bioinformatics (2016) 32:2847–9. doi: 10.1093/bioinformatics/btw313 27207943

[22] Folch J Lees M Sloane Stanley GH. A Simple Method for the Isolation and Purification of Total Lipides From Animal Tissues. J Biol Chem (1957) 226:497–509. doi: 10.1016/S0021-9258(18)64849-5 13428781

[23] Morrison WR Smith LM. Preparation of Fatty Acid Methyl Esters and Dimethylacetals From Lipids With Boron Fluoride–Methanol. J Lipid Res (1964) 5:600–8. doi: 10.1016/S0022-2275(20)40190-7 14221106

[24] Melamud E Vastag L Rabinowitz JD. Metabolomic Analysis and Visualization Engine for LC-MS Data. Anal Chem (2010) 82:9818–26. doi: 10.1021/ac1021166 PMC5748896 21049934

[25] Su X Lu W Rabinowitz JD. Metabolite Spectral Accuracy on Orbitraps. Anal Chem (2017) 89:5940–8. doi: 10.1021/acs.analchem.7b00396 PMC5748891 28471646

[26] Chong J Soufan O Li C Caraus I Li S Bourque G. MetaboAnalyst 4.0: Towards More Transparent and Integrative Metabolomics Analysis. Nucleic Acids Res (2018) 46:W486–94. doi: 10.1093/nar/gky310 PMC6030889 29762782

[27] Xia J Sinelnikov IV Han B Wishart DS. MetaboAnalyst 3.0–Making Metabolomics More Meaningful. Nucleic Acids Res (2015) 43:W251–7. doi: 10.1093/nar/gkv380 PMC4489235 25897128

[28] Diamond TL Roshal M Jamburuthugoda VK Reynolds HM Merriam AR Lee KY. Macrophage Tropism of HIV-1 Depends on Efficient Cellular dNTP Utilization by Reverse Transcriptase. J Biol Chem (2004) 279:51545–53. doi: 10.1074/jbc.M408573200 PMC1351161 15452123

[29] Bolte S Cordelieres FP. A Guided Tour Into Subcellular Colocalization Analysis in Light Microscopy. J Microsc (2006) 224:213–32. doi: 10.1111/j.1365-2818.2006.01706.x 17210054

[30] Rudolph MC Karl Maluf N Wellberg EA Johnson CA Murphy RC Anderson SM. Mammalian Fatty Acid Synthase Activity From Crude Tissue Lysates Tracing (1)(3)C-Labeled Substrates Using Gas Chromatography-Mass Spectrometry. Anal Biochem (2012) 428:158–66. doi: 10.1016/j.ab.2012.06.013 PMC3415257 22728958

[31] Joshi AK Rangan VS Smith S. Differential Affinity Labeling of the Two Subunits of the Homodimeric Animal Fatty Acid Synthase Allows Isolation of Heterodimers Consisting of Subunits That Have Been Independently Modified. J Biol Chem (1998) 273:4937–43. doi: 10.1074/jbc.273.9.4937 9478938

[32] Fornalewicz K Wieczorek A Wegrzyn G Lyzen R. Silencing of the Pentose Phosphate Pathway Genes Influences DNA Replication in Human Fibroblasts. Gene (2017) 635:33–8. doi: 10.1016/j.gene.2017.09.005 28887160

[33] Jing X Wang XJ Zhang T Zhu W Fang Y Wu H. Cell-Cycle-Dependent Phosphorylation of PRPS1 Fuels Nucleotide Synthesis and Promotes Tumorigenesis. Cancer Res (2019) 79:4650–64. doi: 10.1158/0008-5472.CAN-18-2486 31253668

[34] Vizan P Alcarraz-Vizan G Diaz-Moralli S Solovjeva ON Frederiks WM Cascante M. Modulation of Pentose Phosphate Pathway During Cell Cycle Progression in Human Colon Adenocarcinoma Cell Line HT29. Int J Cancer (2009) 124:2789–96. doi: 10.1002/ijc.24262 19253370

[35] Villa E Ali ES Sahu U Ben-Sahra I. Cancer Cells Tune the Signaling Pathways to Empower De Novo Synthesis of Nucleotides. Cancers (Basel) (2019) 11. doi: 10.3390/cancers11050688 PMC6562601 31108873

[36] Diab R Degobert G Hamoudeh M Dumontet C Fessi H. Nucleoside Analogue Delivery Systems in Cancer Therapy. Expert Opin Drug Deliv (2007) 4:513–31. doi: 10.1517/17425247.4.5.513 17880274

[37] Patra KC Hay N. The Pentose Phosphate Pathway and Cancer. Trends Biochem Sci (2014) 39:347–54. doi: 10.1016/j.tibs.2014.06.005 PMC4329227 25037503

[38] Traut TW. Physiological Concentrations of Purines and Pyrimidines. Mol Cell Biochem (1994) 140:1–22. doi: 10.1007/BF00928361 7877593

[39] Louie SM Roberts LS Mulvihill MM Luo K Nomura DK. Cancer Cells Incorporate and Remodel Exogenous Palmitate Into Structural and Oncogenic Signaling Lipids. Biochim Biophys Acta (2013) 1831:1566–72. doi: 10.1016/j.bbalip.2013.07.008 PMC3838875 23872477

[40] Meisenberg G Simmons WH. Principles of Medical Biochemistry Vol. xii. Philadelphia, PA: Elsevier (2017). p. 617.

[41] Dufort FJ Gumina MR Ta NL Tao Y Heyse SA Scott DA. Glucose-Dependent *De Novo* Lipogenesis in B Lymphocytes: A Requirement for Atp-Citrate Lyase in Lipopolysaccharide-Induced Differentiation. J Biol Chem (2014) 289:7011–24. doi: 10.1074/jbc.M114.551051 PMC3945362 24469453

[42] Reitzer LJ Wice BM Kennell D. The Pentose Cycle. Control and Essential Function in HeLa Cell Nucleic Acid Synthesis. J Biol Chem (1980) 255:5616–26. doi: 10.1016/S0021-9258(19)70674-7 6445904

[43] Stincone A Prigione A Cramer T Wamelink MM Campbell K Cheung E. The Return of Metabolism: Biochemistry and Physiology of the Pentose Phosphate Pathway. Biol Rev Camb Philos Soc (2015) 90:927–63. doi: 10.1111/brv.12140 PMC4470864 25243985

[44] Roth EF Jr. Ruprecht RM Schulman S Vanderberg J Olson JA. Ribose Metabolism and Nucleic Acid Synthesis in Normal and Glucose-6-Phosphate Dehydrogenase-Deficient Human Erythrocytes Infected With Plasmodium Falciparum. J Clin Invest (1986) 77:1129–35. doi: 10.1172/JCI112412 PMC424447 2420826

[45] Lane AN Fan TW. Regulation of Mammalian Nucleotide Metabolism and Biosynthesis. Nucleic Acids Res (2015) 43:2466–85. doi: 10.1093/nar/gkv047 PMC4344498 25628363

