# Supplementary figures and images for: Oncogenic Integration of Nucleotide Metabolism via Fatty Acid Synthase in Non-Hodgkin Lymphoma
Source: Front Oncol. 2021 Oct 26;11:725137. doi: 10.3389/fonc.2021.725137 (PMC8576537; doi:10.3389/fonc.2021.725137)

Figure S1

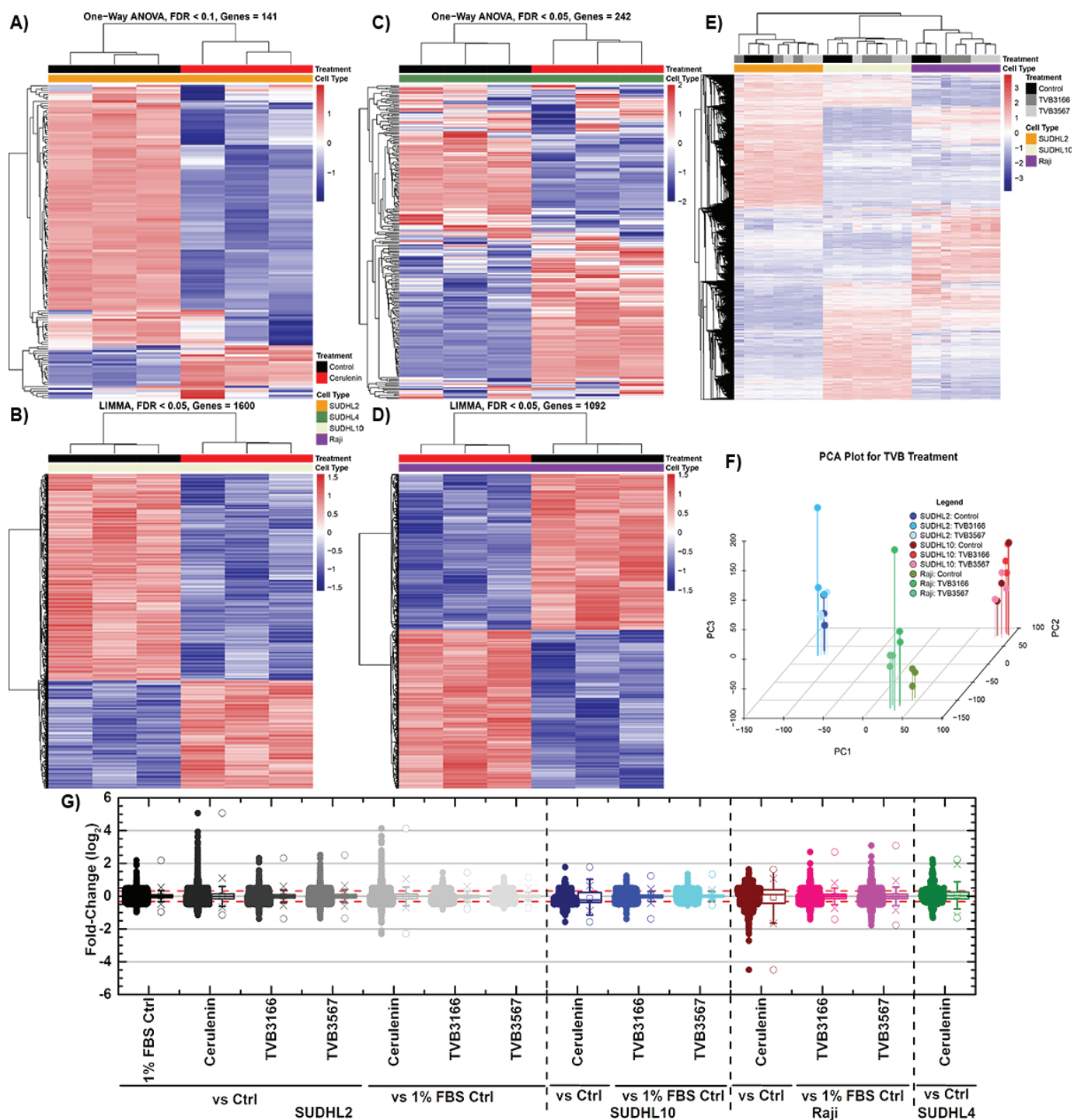

Figure S2

A

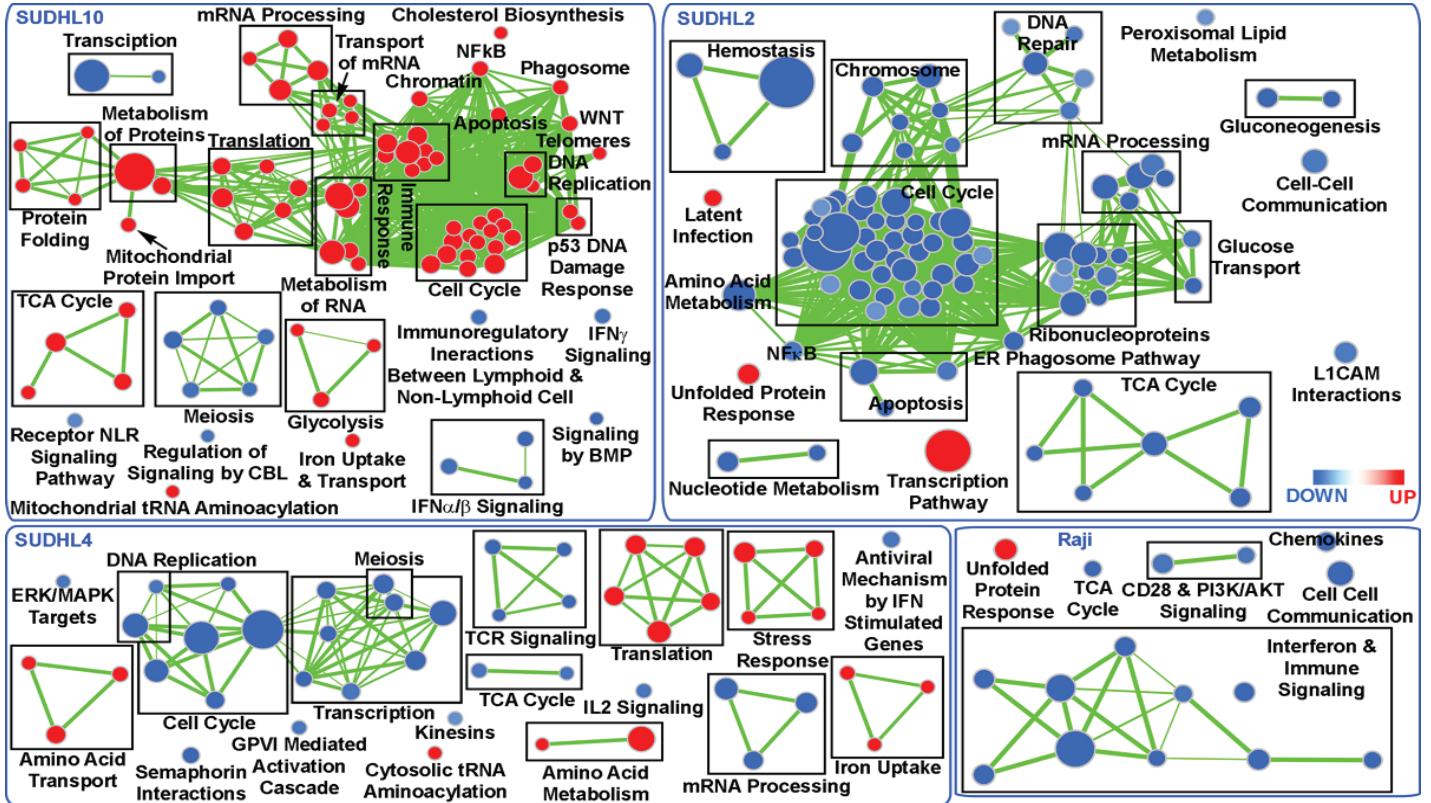

B

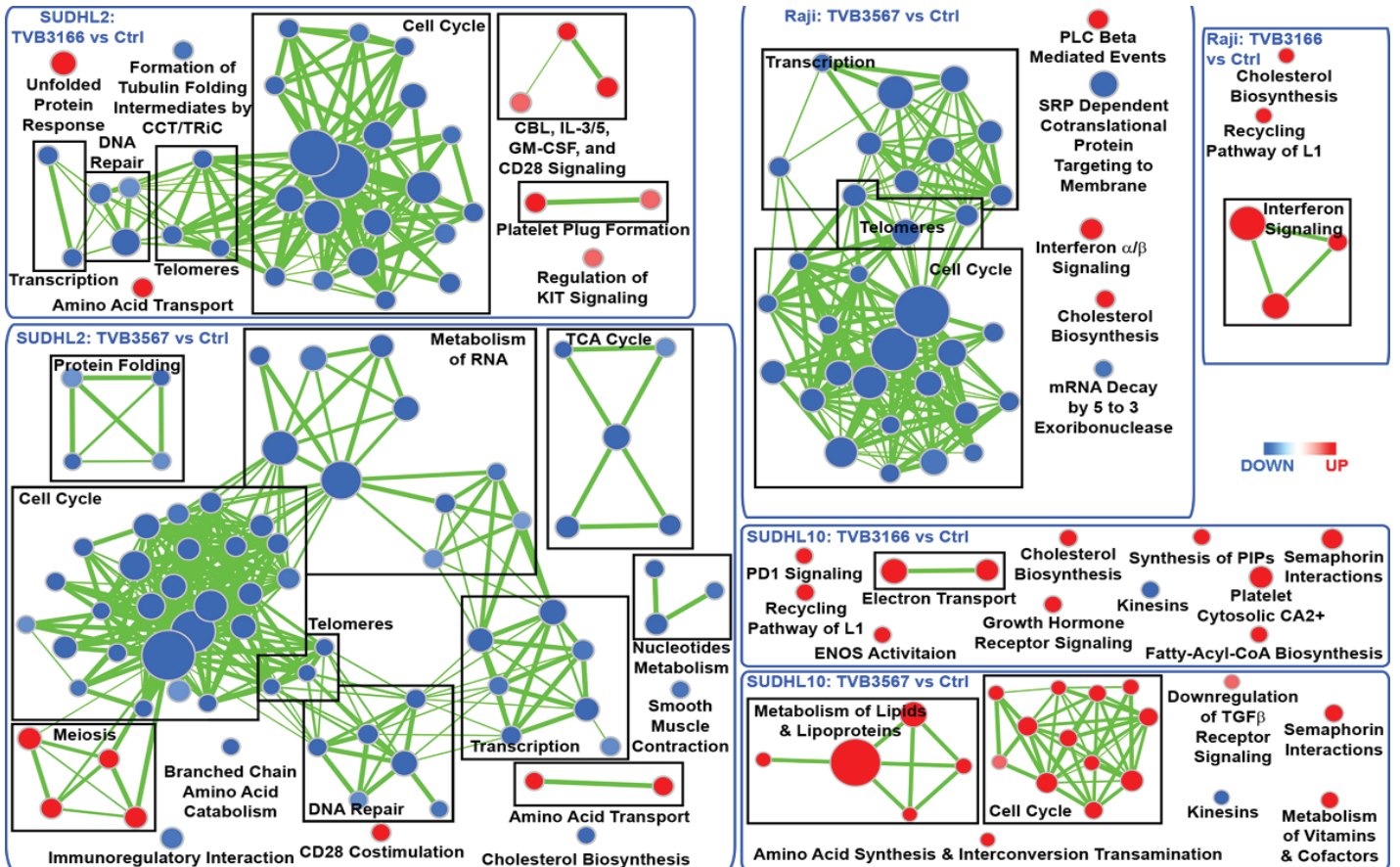

FigureS3

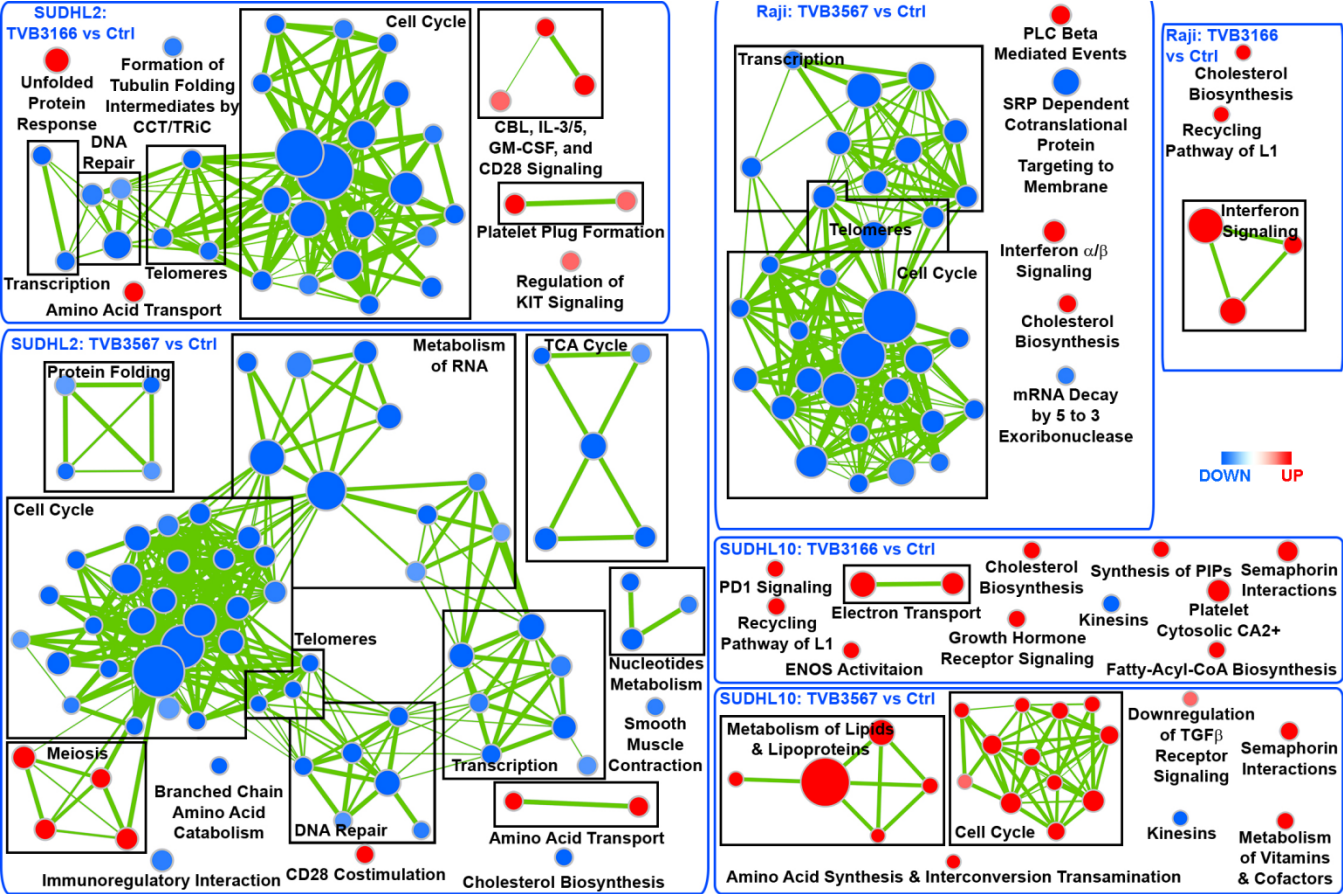

**Figure S4**

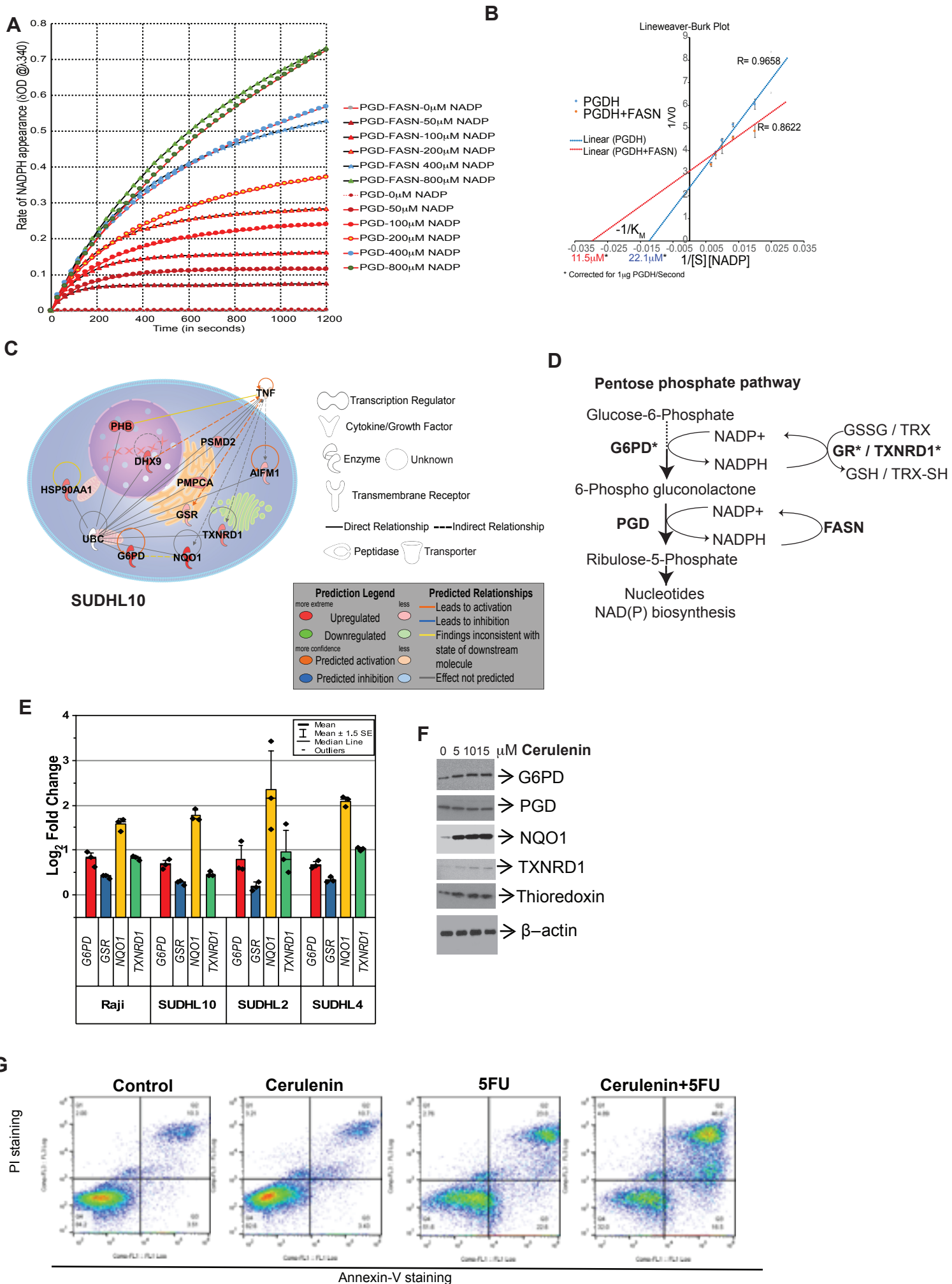

Supplement: Supplementary Figure S1 — Identification of differentially expressed genes by transcriptomic profiling with FASN inhibitors in bNHL cells. Hierarchical clustering of genes and Euclidean distance calculation for 48 hours of 12.5µM cerulenin treatment on (A) SUDHL2 cells with One-Way ANOVA, FDR < 0.1 (141 genes), (B) SUDHL10 cells with LIMMA analysis, FDR < 0.05 (1600 genes) (C) SUDHL4 cells with One-Way ANOVA, FDR < 0.05 (242 genes), (D) Raji cells with LIMMA analysis, FDR < 0.05 (1092 genes). (E) Hierarchical clustering of genes and Euclidean distance calculation for 48 hours of TVB3166 or 3657 in SUDHL2, SUDHL10 and Raji cells with One-Way ANOVA, FDR < 0.05 (8872 genes). (F) Principal component analysis plot of TVB3166 or TVB3567 treated bNHL cells show global differences between the experimental conditions and the untreated controls for SUDHL2, SUDHL10 and Raji cells. (G) Scatter plot represents fold changes in the gene expression from the transcriptome of TVB3166 or 3657 or cerlulenin treated bNHL cells (no significant difference were detected in the gene expression pattern with cerulenin treatment performed in the presence of 1% or 10% serum containing medium, data not shown), with whiskers showing the range of the outliers, with max and min values as O and the 1 and 99th percentile outliers as X. Individual data points are shown on the left of box plots as filled circles. Dotted red lines show the 1.2 log2 fold-change cutoff. [file DataSheet_1.pdf]
