# Supplementary material for: Oncogenic Integration of Nucleotide Metabolism via Fatty Acid Synthase in Non-Hodgkin Lymphoma
Source: Front Oncol. 2021 Oct 26;11:725137. doi: 10.3389/fonc.2021.725137 (PMC8576537; doi:10.3389/fonc.2021.725137)
Supplement: Supplementary Table S1 — Pathway enrichment by multi-omic analysis comparing cerulenin transcriptome and metabolome. List of pathways determined using significant genes and metabolites from cerulenin treated SUDHL10 cells analyzed based on pathway centrality and degree of enrichment for determination of high impact pathways shown in Figure 2E, is included in this table. Impact scores, FDR, P values, number of molecules and hits are included in this table. [file DataSheet_2.pdf]

Biological Pathways	Total	Expected	Hits	Raw p	LOG10(p)	Holm adjust	FDR	Impact
Synthesis and degradation of ketone bodies	16	1.0166	6	0.00029728	3.5268	0.087699	0.0026595	3.7
Purine metabolism	225	14.295	28	0.00047959	3.3191	0.13812	0.0036078	2.1782
Citrate cycle (TCA cycle)	50	3.1768	14	1.69E-06	5.7715	0.00053992	4.17E-05	1.9318
Fatty acid biosynthesis	76	4.8287	4	0.72001	0.14266	1	0.91663	1.8837
Pyruvate metabolism	70	4.4475	13	0.00040208	3.3957	0.11741	0.003246	1.7925
Sphingolipid metabolism	72	4.5746	6	0.30707	0.51277	1	0.50404	1.7541
Pyrimidine metabolism	122	7.7513	18	0.00066967	3.1741	0.19153	0.0048187	1.5659
Cell cycle	124	7.8784	32	4.38E-12	11.359	1.44E-09	7.25E-10	1.5455
JAK-STAT signaling pathway	162	10.293	9	0.70889	0.14942	1	0.90946	1.5278
Glycolysis or Gluconeogenesis	99	6.29	13	0.0096723	2.0145	1	0.036799	1.3846
Phosphatidylinositol signaling system	128	8.1325	9	0.42657	0.37001	1	0.63889	1.3506
One carbon pool by folate	29	1.8425	2	0.55807	0.25331	1	0.77264	1.3333
Valine, leucine and isoleucine degradation	90	5.7182	12	0.01126	1.9484	1	0.040513	1.1667
Glycerophospholipid metabolism	149	9.4668	13	0.1523	0.81731	1	0.30185	1.1333
Alanine, aspartate and glutamate metabolism	64	4.0663	15	8.20E-06	5.0862	0.0025419	0.00011808	1.1148
Glutathione metabolism	94	5.9723	16	0.00025826	3.5879	0.076961	0.0024264	1.0725
Fatty acid degradation	94	5.9723	4	0.85612	0.067467	1	1	1.0672
Steroid biosynthesis	77	4.8922	8	0.11465	0.94064	1	0.24803	1.051
Pentose phosphate pathway	65	4.1298	10	0.0075066	2.1246	1	0.031855	1.0476
Nicotinate and nicotinamide metabolism	92	5.8453	11	0.031039	1.5081	1	0.092558	1.037
Central carbon metabolism in cancer	106	6.7348	31	2.47E-13	12.608	8.17E-11	8.17E-11	0.23009
Epstein-Barr virus infection	204	12.961	39	4.11E-10	9.3865	1.35E-07	4.53E-08	0.69388
Cellular senescence	165	10.483	33	2.83E-09	8.5482	9.28E-07	2.34E-07	0.7265
Measles	139	8.8314	29	9.55E-09	8.02	3.12E-06	6.32E-07	0.55056
p53 signaling pathway	72	4.5746	20	1.19E-08	7.9228	3.89E-06	6.59E-07	0.36364
Hepatitis B	163	10.356	30	1.07E-07	6.9718	3.47E-05	5.05E-06	0.47826
DNA replication	36	2.2873	13	1.46E-07	6.8364	4.72E-05	6.03E-06	0
Protein processing in endoplasmic reticulum	172	10.928	30	3.63E-07	6.4406	0.00011711	1.33E-05	0.16981
Proteasome	45	2.8591	14	4.06E-07	6.392	0.00013058	1.34E-05	0
Endocytosis	255	16.202	38	6.98E-07	6.1559	0.00022418	2.10E-05	0.12409
Ubiquitin mediated proteolysis	136	8.6408	25	1.25E-06	5.9018	0.00040124	3.46E-05	0
Human T-cell leukemia virus 1 infection	224	14.232	34	1.76E-06	5.7537	0.00056069	4.17E-05	0.36923
Alzheimer disease	178	11.309	29	2.41E-06	5.6185	0.00076299	5.17E-05	0.14925
Glucagon signaling pathway	132	8.3867	24	2.50E-06	5.6024	0.00078943	5.17E-05	0.36145
Non-alcoholic fatty liver disease (NAFLD)	151	9.5938	26	2.79E-06	5.5551	0.00087749	5.42E-05	0.27143
Viral carcinogenesis	201	12.771	31	3.58E-06	5.4461	0.0011241	6.58E-05	0.0072993
FoxO signaling pathway	136	8.6408	24	4.30E-06	5.3661	0.0013472	7.50E-05	0.84524
Parkinson disease	157	9.9751	26	5.85E-06	5.2329	0.0018249	9.68E-05	0.1194
RNA transport	168	10.674	27	6.93E-06	5.1591	0.0021563	0.00010928	0.3945
Insulin signaling pathway	141	8.9585	24	8.20E-06	5.0859	0.0025419	0.00011808	0.69697
HIF-1 signaling pathway	124	7.8784	21	3.22E-05	4.4918	0.0099255	0.00044445	0.84146
Huntington disease	196	12.453	28	4.44E-05	4.3529	0.013621	0.00058745	0.10294
Oxidative phosphorylation	149	9.4668	23	6.28E-05	4.202	0.019221	0.00079966	0.021739
Thyroid cancer	37	2.3508	10	7.29E-05	4.1374	0.022229	0.00089347	0.76
Ferroptosis	71	4.511	14	0.00012509	3.9028	0.038028	0.0014788	0.24324
Nucleotide excision repair	47	2.9862	11	0.00013338	3.8749	0.040414	0.0015224	0
Renal cell carcinoma	72	4.5746	14	0.00014638	3.8345	0.044207	0.0016151	0.59649
MAPK signaling pathway	300	19.061	36	0.0001662	3.7794	0.050025	0.0017745	0.82258
Apoptosis	140	8.895	21	0.0001955	3.7089	0.058649	0.0020041	0.51327
Glyoxylate and dicarboxylate metabolism	92	5.8453	16	0.00019981	3.6994	0.059743	0.0020041	0.46591
Human immunodeficiency virus 1 infection	217	13.787	28	0.00026388	3.5786	0.078374	0.0024264	0.43284
AMPK signaling pathway	143	9.0856	21	0.0002639	3.5786	0.078374	0.0024264	0.1383
Chronic myeloid leukemia	77	4.8922	14	0.00030634	3.5138	0.090064	0.0026684	0.49091
Colorectal cancer	87	5.5276	15	0.00034545	3.4616	0.10122	0.0029319	0.45946
Butanoate metabolism	70	4.4475	13	0.00040208	3.3957	0.11741	0.003246	0.98182
Prostate cancer	108	6.8618	17	0.00043762	3.3589	0.12691	0.0034293	0.48387
Kaposi sarcoma-associated herpesvirus infection	191	12.135	25	0.0004455	3.3511	0.12875	0.0034293	0.38346
Oocyte meiosis	132	8.3867	19	0.00065648	3.1828	0.18841	0.0048187	0.50588
Neurotrophin signaling pathway	124	7.8784	18	0.00081469	3.089	0.23219	0.0057375	0.60976
Base excision repair	33	2.0967	8	0.00085492	3.0681	0.2428	0.0058663	0
Spliceosome	135	8.5773	19	0.00086843	3.0613	0.24577	0.0058663	0
Small cell lung cancer	95	6.0359	15	0.00090309	3.0443	0.25467	0.0059785	0.5098
Hepatitis C	157	9.9751	21	0.00093657	3.0285	0.26318	0.0060785	0.45361
C-type lectin receptor signaling pathway	116	7.3701	17	0.0010098	2.9958	0.28274	0.0064278	0.32105
AGE-RAGE signaling pathway in diabetic complications	109	6.9254	16	0.0013807	2.8599	0.38522	0.008623	0.83824
Gastric cancer	153	9.7209	20	0.0016295	2.7879	0.45301	0.0099885	0.50485
NOD-like receptor signaling pathway	187	11.881	23	0.0017359	2.7605	0.48085	0.010447	0.30636
TNF signaling pathway	112	7.116	16	0.0018449	2.734	0.5092	0.010905	0.29897
Fanconi anemia pathway	54	3.4309	10	0.00188	2.7258	0.51701	0.010917	0.25926
mTOR signaling pathway	157	9.9751	20	0.0022268	2.6523	0.61016	0.012656	0.36905
Human papillomavirus infection	333	21.157	35	0.002256	2.6467	0.61588	0.012656	0.51875
IL-17 signaling pathway	94	5.9723	14	0.0023409	2.6306	0.63674	0.012914	0.055556
Thyroid hormone synthesis	95	6.0359	14	0.0025895	2.5868	0.70174	0.014051	0.063158
Hepatocellular carcinoma	182	11.563	22	0.0026929	2.5698	0.72709	0.014377	0.5
Thermogenesis	254	16.138	28	0.003058	2.5146	0.8226	0.016067	0.20482
Longevity regulating pathway	97	6.1629	14	0.0031518	2.5014	0.84467	0.016109	0.53704
Influenza A	173	10.992	21	0.0031635	2.4998	0.84467	0.016109	0.30702
Transcriptional misregulation in cancer	186	11.818	22	0.003524	2.453	0.93739	0.017621	0

Pancreatic cancer	78	4.9558	12	0.0035669	2.4477	0.94522	0.017621	0.49123
Endometrial cancer	59	3.7486	10	0.0037082	2.4308	0.97896	0.01805	0.43478
Shigellosis	69	4.3839	11	0.0039097	2.4079	1	0.018755	0.38095
Progesterone-mediated oocyte maturation	103	6.5441	14	0.0054632	2.2626	1	0.025658	0.375
Fluid shear stress and atherosclerosis	147	9.3397	18	0.0055037	2.2593	1	0.025658	0.46667
Arginine biosynthesis	44	2.7956	8	0.0058858	2.2302	1	0.027058	0.62857
Pathways in cancer	562	35.707	51	0.0060567	2.2178	1	0.02712	0.4321
Melanoma	73	4.6381	11	0.006063	2.2173	1	0.02712	0.63636
Long-term potentiation	74	4.7016	11	0.0067249	2.1723	1	0.029485	0.51613
Basal transcription factors	45	2.8591	8	0.0067699	2.1694	1	0.029485	0
Mitophagy - animal	65	4.1298	10	0.0075066	2.1246	1	0.031855	0.32653
Aminoacyl-tRNA biosynthesis	118	7.4972	15	0.0076705	2.1152	1	0.032139	0.26804
B cell receptor signaling pathway	86	5.464	12	0.0079124	2.1017	1	0.032738	0.46
T cell receptor signaling pathway	108	6.8618	14	0.008292	2.0813	1	0.033884	0.5493
Choline metabolism in cancer	109	6.9254	14	0.0089765	2.0469	1	0.036106	0.30189
Prion diseases	38	2.4143	7	0.0090902	2.0414	1	0.036106	0.17188
Autophagy - animal	143	9.0856	17	0.009163	2.038	1	0.036106	0.28333
Retrograde endocannabinoid signaling	167	10.61	19	0.0096396	2.0159	1	0.036799	0.2619
Gap junction	99	6.29	13	0.0096723	2.0145	1	0.036799	0.32692
ErbB signaling pathway	89	5.6547	12	0.010336	1.9856	1	0.038879	0.3913
Th17 cell differentiation	111	7.0524	14	0.010479	1.9797	1	0.038972	0.41905
Glioma	79	5.0193	11	0.010923	1.9617	1	0.039731	0.56164
RNA degradation	79	5.0193	11	0.010923	1.9617	1	0.039731	0.016129
RNA polymerase	31	1.9696	6	0.012095	1.9174	1	0.043047	0
Protein export	23	1.4613	5	0.01318	1.8801	1	0.045922	0
Mismatch repair	23	1.4613	5	0.01318	1.8801	1	0.045922	0
Homologous recombination	41	2.605	7	0.01374	1.862	1	0.047373	0.1
Proteoglycans in cancer	211	13.406	22	0.014954	1.8253	1	0.051027	0.28
Non-small cell lung cancer	72	4.5746	10	0.015121	1.8204	1	0.051073	0.44643
EGFR tyrosine kinase inhibitor resistance	83	5.2734	11	0.015532	1.8088	1	0.051929	0.51515
Bacterial invasion of epithelial cells	73	4.6381	10	0.016559	1.781	1	0.054811	0.55814
Osteoclast differentiation	131	8.3231	15	0.018988	1.7215	1	0.062099	0.47368
Cytosolic DNA-sensing pathway	64	4.0663	9	0.019136	1.7181	1	0.062099	0.25806
Pathogenic Escherichia coli infection	204	12.961	21	0.019528	1.7093	1	0.062756	0.48
Aldosterone synthesis and secretion	120	7.6243	14	0.019811	1.7031	1	0.063053	0.24638
VEGF signaling pathway	65	4.1298	9	0.021005	1.6777	1	0.065669	0.42424
Regulation of actin cytoskeleton	218	13.851	22	0.02103	1.6772	1	0.065669	0.59036
Mineral absorption	87	5.5276	11	0.021466	1.6682	1	0.066405	0.035294
Terpenoid backbone biosynthesis	67	4.2569	9	0.025131	1.5998	1	0.077023	0.69355
Acute myeloid leukemia	68	4.3204	9	0.027396	1.5623	1	0.083194	0.45833
Pantothenate and CoA biosynthesis	47	2.9862	7	0.027723	1.5572	1	0.08342	0.66667
Prolactin signaling pathway	81	5.1464	10	0.031915	1.496	1	0.093406	0.23077
Toll-like receptor signaling pathway	104	6.6077	12	0.031916	1.496	1	0.093406	0.42857
cGMP-PKG signaling pathway	177	11.246	18	0.032333	1.4904	1	0.093406	0.21951
Insulin resistance	128	8.1325	14	0.032452	1.4888	1	0.093406	0.21324
PD-L1 expression and PD-1 checkpoint pathway in cancer	93	5.9088	11	0.033269	1.478	1	0.094931	0.5
Human cytomegalovirus infection	231	14.677	22	0.0372	1.4295	1	0.10524	0.29091
Amyotrophic lateral sclerosis (ALS)	61	3.8757	8	0.038189	1.4181	1	0.10712	0.22222
Proximal tubule bicarbonate reclamation	40	2.5414	6	0.039001	1.4089	1	0.10848	0
Bladder cancer	41	2.605	6	0.043332	1.3632	1	0.11952	0.40625
Sphingolipid signaling pathway	134	8.5137	14	0.045192	1.3449	1	0.12282	0.2561
Non-homologous end-joining	13	0.82596	3	0.045268	1.3442	1	0.12282	0
Necroptosis	172	10.928	17	0.046226	1.3351	1	0.1244	0.34043
GnRH signaling pathway	99	6.29	11	0.049058	1.3093	1	0.13095	0.29787
Yersinia infection	125	7.9419	13	0.053885	1.2685	1	0.14269	0.58333
Taurine and hypotaurine metabolism	33	2.0967	5	0.05533	1.257	1	0.14535	0
Selenocompound metabolism	44	2.7956	6	0.058072	1.236	1	0.15135	0.7619
Signaling pathways regulating pluripotency of stem cells	140	8.895	14	0.061088	1.214	1	0.15797	0.32653
mRNA surveillance pathway	91	5.7817	10	0.062501	1.2041	1	0.16037	0.42857
Taste transduction	116	7.3701	12	0.064366	1.1913	1	0.16389	0.35211
Cushing syndrome	168	10.674	16	0.068126	1.1667	1	0.17214	0.34483
Vasopressin-regulated water reabsorption	46	2.9226	6	0.069376	1.1588	1	0.17397	0.074074
Long-term depression	69	4.3839	8	0.070119	1.1542	1	0.17451	0.3913
RIG-I-like receptor signaling pathway	70	4.4475	8	0.074992	1.125	1	0.18436	0.47368
Propanoate metabolism	82	5.2099	9	0.075194	1.1238	1	0.18436	0.70833
Endocrine resistance	108	6.8618	11	0.081139	1.0908	1	0.19604	0.44286
Chagas disease (American trypanosomiasis)	108	6.8618	11	0.081139	1.0908	1	0.19604	0.41096
Cholesterol metabolism	60	3.8121	7	0.084678	1.0722	1	0.20164	0.125
Viral myocarditis	60	3.8121	7	0.084678	1.0722	1	0.20164	0.12121
Natural killer cell mediated cytotoxicity	135	8.5773	13	0.087393	1.0585	1	0.20662	0.26531
Primary immunodeficiency	38	2.4143	5	0.090655	1.0426	1	0.21174	0
Growth hormone synthesis, secretion and action	123	7.8149	12	0.090836	1.0417	1	0.21174	0.4127
Herpes simplex virus 1 infection	494	31.386	39	0.09267	1.0331	1	0.2145	0.34091
Cysteine and methionine metabolism	112	7.116	11	0.098809	1.0052	1	0.22564	0.54369
Allograft rejection	39	2.4779	5	0.098845	1.005	1	0.22564	0.16667
D-Glutamine and D-glutamate metabolism	18	1.1436	3	0.10233	0.99	1	0.2314	0.375
Hedgehog signaling pathway	51	3.2403	6	0.10277	0.98815	1	0.2314	0.21429
Phagosome	153	9.7209	14	0.10735	0.96919	1	0.24009	0.064103
Platinum drug resistance	76	4.8287	8	0.1084	0.96499	1	0.2408	0.34

Longevity regulating pathway - multiple species	64	4.0663	7	0.11018	0.95789	1	0.24314	0.4717
Hippo signaling pathway	154	9.7845	14	0.1116	0.95233	1	0.24464	0.32353
Toxoplasmosis	115	7.3066	11	0.11345	0.9452	1	0.24705	0.29508
Antigen processing and presentation	78	4.9558	8	0.12109	0.9169	1	0.25858	0.34211
Amphetamine addiction	78	4.9558	8	0.12109	0.9169	1	0.25858	0.22115
Dopaminergic synapse	143	9.0856	13	0.12193	0.91389	1	0.25871	0.41667
NF-kappa B signaling pathway	105	6.6712	10	0.12972	0.887	1	0.27175	0.33129
Ribosome biogenesis in eukaryotes	105	6.6712	10	0.12972	0.887	1	0.27175	0
Graft-versus-host disease	43	2.732	5	0.13509	0.86938	1	0.28093	0.17391
Apelin signaling pathway	146	9.2762	13	0.13665	0.8644	1	0.28093	0.42667
Estrogen signaling pathway	146	9.2762	13	0.13665	0.8644	1	0.28093	0.28571
Apoptosis - multiple species	32	2.0331	4	0.14265	0.84572	1	0.29147	0.36364
Wnt signaling pathway	161	10.229	14	0.14409	0.84137	1	0.29259	0.35366
Inositol phosphate metabolism	121	7.6878	11	0.14622	0.835	1	0.29511	0.95294
Leishmaniasis	82	5.2099	8	0.14869	0.82771	1	0.29829	0.27273
Relaxin signaling pathway	135	8.5773	12	0.14968	0.82482	1	0.29847	0.53333
Circadian rhythm	33	2.0967	4	0.1547	0.81051	1	0.30479	0.25
Breast cancer	150	9.5303	13	0.15771	0.80213	1	0.30889	0.35
Oxytocin signaling pathway	165	10.483	14	0.16474	0.78321	1	0.32054	0.26316
Type I diabetes mellitus	46	2.9226	5	0.16559	0.78095	1	0.32054	0.095238
Platelet activation	138	8.7679	12	0.16695	0.77742	1	0.32128	0.35632
Pertussis	86	5.464	8	0.17906	0.747	1	0.34063	0.17647
Renin secretion	86	5.464	8	0.17906	0.747	1	0.34063	0.071429
Arginine and proline metabolism	128	8.1325	11	0.18996	0.72135	1	0.35929	0.48718
Tight junction	170	10.801	14	0.19255	0.71546	1	0.36076	0.46667
Nitrogen metabolism	36	2.2873	4	0.19292	0.71463	1	0.36076	0.13043
Parathyroid hormone synthesis, secretion and action	116	7.3701	10	0.20187	0.69494	1	0.37538	0.21951
Thyroid hormone signaling pathway	130	8.2596	11	0.20344	0.69156	1	0.3762	0.24528
Rap1 signaling pathway	215	13.66	17	0.20709	0.68385	1	0.38081	0.43617
beta-Alanine metabolism	63	4.0027	6	0.20957	0.67868	1	0.38113	0.74074
Phosphonate and phosphinate metabolism	63	4.0027	6	0.20957	0.67868	1	0.38113	0.15789
Phospholipase D signaling pathway	159	10.102	13	0.21073	0.67628	1	0.38115	0.42254
Glycine, serine and threonine metabolism	90	5.7182	8	0.2119	0.67388	1	0.38118	0.68235
Galactose metabolism	77	4.8922	7	0.21632	0.6649	1	0.38704	0.28169
Calcium signaling pathway	203	12.898	16	0.21956	0.65844	1	0.38914	0.33824
Basal cell carcinoma	64	4.0663	6	0.21985	0.65788	1	0.38914	0.32
Intestinal immune network for IgA production	51	3.2403	5	0.22163	0.65437	1	0.39021	0.055556
Valine, leucine and isoleucine biosynthesis	27	1.7155	3	0.24413	0.61239	1	0.42754	0.30769
TGF-beta signaling pathway	94	5.9723	8	0.24684	0.60759	1	0.43001	0.6625
Chemokine signaling pathway	194	12.326	15	0.25143	0.59958	1	0.43573	0.57812
Riboflavin metabolism	28	1.779	3	0.26158	0.58239	1	0.45096	0.20833
Cholinergic synapse	124	7.8784	10	0.26298	0.58008	1	0.45102	0.29508
ABC transporters	183	11.627	14	0.27386	0.56247	1	0.46606	0
Peroxisome	83	5.2734	7	0.27456	0.56136	1	0.46606	0
GABAergic synapse	98	6.2265	8	0.28349	0.54747	1	0.47828	0.28571
Salmonella infection	84	5.337	7	0.28466	0.54568	1	0.47828	0.38776
Drug metabolism - other enzymes	142	9.022	11	0.2919	0.53476	1	0.48798	0.58696
Autoimmune thyroid disease	57	3.6215	5	0.29496	0.53023	1	0.48953	0.073171
Adherens junction	71	4.511	6	0.29579	0.52902	1	0.48953	0.14667
Legionellosis	58	3.6851	5	0.3076	0.51201	1	0.50404	0.0625
Starch and sucrose metabolism	73	4.6381	6	0.31842	0.497	1	0.51468	0.52632
GnRH secretion	73	4.6381	6	0.31842	0.497	1	0.51468	0.17021
Aldosterone-regulated sodium reabsorption	45	2.8591	4	0.32001	0.49484	1	0.51468	0.28571
Vibrio cholerae infection	59	3.7486	5	0.32031	0.49442	1	0.51468	0.071429
MicroRNAs in cancer	310	19.696	22	0.32552	0.48743	1	0.52051	0.10022
PI3K-Akt signaling pathway	358	22.746	25	0.34031	0.46813	1	0.54154	0.39216
Sulfur relay system	19	1.2072	2	0.34239	0.46547	1	0.54226	0
Endocrine and other factor-regulated calcium reabsorption	61	3.8757	5	0.3459	0.46105	1	0.54521	0.14286
Thiamine metabolism	47	2.9862	4	0.34945	0.45662	1	0.54818	0.10526
Epithelial cell signaling in Helicobacter pylori infection	77	4.8922	6	0.36434	0.4385	1	0.56617	0.19048
Cortisol synthesis and secretion	77	4.8922	6	0.36434	0.4385	1	0.56617	0.13559
SNARE interactions in vesicular transport	34	2.1602	3	0.36799	0.43417	1	0.56653	0.15789
Renin-angiotensin system	34	2.1602	3	0.36799	0.43417	1	0.56653	0.025641
Tuberculosis	197	12.516	14	0.37162	0.4299	1	0.56753	0.27338
Axon guidance	182	11.563	13	0.37207	0.42938	1	0.56753	0.22819
Serotonergic synapse	157	9.9751	11	0.41361	0.38341	1	0.628	0.30769
Lysosome	127	8.069	9	0.41736	0.37949	1	0.6308	0
Type II diabetes mellitus	52	3.3038	4	0.4228	0.37386	1	0.63612	0.35484
Insulin secretion	98	6.2265	7	0.43129	0.36523	1	0.64305	0.12281
Amoebiasis	115	7.3066	8	0.44852	0.34822	1	0.66574	0.066667
Ras signaling pathway	239	15.185	16	0.45158	0.34527	1	0.66729	0.58333
Leukocyte transendothelial migration	116	7.3701	8	0.45827	0.33888	1	0.67417	0.27473
Histidine metabolism	70	4.4475	5	0.46092	0.33638	1	0.67481	0.14516
Fc gamma R-mediated phagocytosis	101	6.4171	7	0.46279	0.33462	1	0.67481	0.39286
Pancreatic secretion	117	7.4336	8	0.46799	0.32976	1	0.67941	0.077778
Cocaine addiction	57	3.6215	4	0.49397	0.3063	1	0.71399	0.17284
Synaptic vesicle cycle	90	5.7182	6	0.51246	0.29034	1	0.73607	0.029412
Focal adhesion	201	12.771	13	0.51503	0.28817	1	0.73607	0.80645
Glutamatergic synapse	122	7.7513	8	0.51592	0.28742	1	0.73607	0.34286

Sulfur metabolism	43	2.732	3	0.52035	0.28371	1	0.73755	0.21053
Antifolate resistance	59	3.7486	4	0.52141	0.28282	1	0.73755	0.033333
Hypertrophic cardiomyopathy (HCM)	93	5.9088	6	0.54503	0.26358	1	0.76281	0.065217
D-Arginine and D-ornithine metabolism	12	0.76243	1	0.5453	0.26336	1	0.76281	0
Phenylalanine metabolism	77	4.8922	5	0.54618	0.26266	1	0.76281	0.11111
Protein digestion and absorption	142	9.022	9	0.5525	0.25767	1	0.76839	0
Adrenergic signaling in cardiomyocytes	159	10.102	10	0.56022	0.25164	1	0.77264	0.26761
Fc epsilon RI signaling pathway	79	5.0193	5	0.56936	0.24462	1	0.78198	0.2037
Rheumatoid arthritis	96	6.0994	6	0.57659	0.23913	1	0.78864	0.058824
Inflammatory bowel disease (IBD)	65	4.1298	4	0.59909	0.22251	1	0.81604	0.12903
Lysine degradation	115	7.3066	7	0.60194	0.22045	1	0.81656	0.19481
Glycerolipid metabolism	99	6.29	6	0.60702	0.21679	1	0.81677	0.65517
Dilated cardiomyopathy (DCM)	99	6.29	6	0.60702	0.21679	1	0.81677	0.12766
Inflammatory mediator regulation of TRP channels	135	8.5773	8	0.63217	0.19917	1	0.84716	0.13483
Neomycin, kanamycin and gentamicin biosynthesis	86	5.464	5	0.6453	0.19024	1	0.8559	0.047619
Morphine addiction	103	6.5441	6	0.64566	0.18999	1	0.8559	0.23
Regulation of lipolysis in adipocytes	69	4.3839	4	0.64645	0.18947	1	0.8559	0.1
Lipoic acid metabolism	17	1.0801	1	0.67266	0.1722	1	0.8817	0.42105
Pentose and glucuronate interconversions	89	5.6547	5	0.67514	0.1706	1	0.8817	0.044118
Fat digestion and absorption	54	3.4309	3	0.67577	0.1702	1	0.8817	0
Ether lipid metabolism	72	4.5746	4	0.67945	0.16784	1	0.8817	0.93023
Melanogenesis	107	6.7983	6	0.68192	0.16627	1	0.8817	0.15909
Salivary secretion	107	6.7983	6	0.68192	0.16627	1	0.8817	0.071429
Carbohydrate digestion and absorption	74	4.7016	4	0.70022	0.15476	1	0.90184	0.032787
Glycosylphosphatidylinositol (GPI)-anchor biosynthesis	39	2.4779	2	0.71869	0.14346	1	0.91663	0.39535
Th1 and Th2 cell differentiation	95	6.0359	5	0.72967	0.13687	1	0.92537	0.23944
Tyrosine metabolism	114	7.243	6	0.73933	0.13116	1	0.93404	0.1371
Arrhythmogenic right ventricular cardiomyopathy (ARVC)	79	5.0193	4	0.74785	0.12619	1	0.94121	0
Hematopoietic cell lineage	98	6.2265	5	0.75432	0.12244	1	0.94576	0
PPAR signaling pathway	81	5.1464	4	0.76521	0.11622	1	0.95578	0.13559
Vitamin digestion and absorption	63	4.0027	3	0.77266	0.11201	1	0.96147	0
Amino sugar and nucleotide sugar metabolism	156	9.9115	8	0.78237	0.10659	1	0.9699	0.21384
Alcoholism	194	12.326	10	0.79594	0.099118	1	0.98305	0.29
Mucin type O-glycan biosynthesis	46	2.9226	2	0.79942	0.097228	1	0.98367	0.33333
Fatty acid elongation	67	4.2569	3	0.80744	0.09289	1	0.98787	0.58228
Nicotine addiction	47	2.9862	2	0.8091	0.091996	1	0.98787	0.078125
Circadian entrainment	106	6.7348	5	0.81178	0.090561	1	0.98787	0.46552
Caffeine metabolism	27	1.7155	1	0.83043	0.080698	1	1	0
Cardiac muscle contraction	90	5.7182	4	0.83195	0.0799	1	1	0.023256
Cell adhesion molecules (CAMs)	147	9.3397	7	0.83298	0.079364	1	1	0.079245
Vascular smooth muscle contraction	148	9.4032	7	0.83803	0.076738	1	1	0.25352
Hippo signaling pathway - multiple species	29	1.8425	1	0.85134	0.069897	1	1	0.093023
Notch signaling pathway	53	3.3674	2	0.85891	0.06605	1	1	0.074074
Adipocytokine signaling pathway	76	4.8287	3	0.86954	0.060711	1	1	0.11628
Ascorbate and aldarate metabolism	76	4.8287	3	0.86954	0.060711	1	1	0.018868
Glycosaminoglycan biosynthesis	32	2.0331	1	0.87798	0.056516	1	1	0
Cytokine-cytokine receptor interaction	294	18.679	14	0.89953	0.045985	1	1	0.24249
Autophagy - other	35	2.2237	1	0.89985	0.04583	1	1	0.083333
Collecting duct acid secretion	35	2.2237	1	0.89985	0.04583	1	1	0
cAMP signaling pathway	241	15.312	11	0.90583	0.042955	1	1	0.17213
Phototransduction	36	2.2873	1	0.90623	0.04276	1	1	0
Glycosphingolipid biosynthesis - globo and isoglobo series	38	2.4143	1	0.91781	0.037249	1	1	0.17949
Primary bile acid biosynthesis	64	4.0663	2	0.92057	0.035945	1	1	0.15625
Fructose and mannose metabolism	87	5.5276	3	0.92096	0.03576	1	1	0.13333
Phenylalanine, tyrosine and tryptophan biosynthesis	40	2.5414	1	0.92795	0.032475	1	1	0
Gastric acid secretion	89	5.6547	3	0.92803	0.032439	1	1	0.096154
Ribosome	153	9.7209	6	0.92948	0.031762	1	1	0
African trypanosomiasis	45	2.8591	1	0.94818	0.023111	1	1	0.027778
Ovarian steroidogenesis	73	4.6381	2	0.95111	0.021768	1	1	0.088235
Glycosphingolipid biosynthesis - ganglio series	46	2.9226	1	0.95148	0.0216	1	1	0.32759
Viral protein interaction with cytokine and cytokine receptor	100	6.3535	3	0.95754	0.018843	1	1	0.039474
Mannose type O-glycan biosynthesis	50	3.1768	1	0.96273	0.016496	1	1	0.020833
Malaria	54	3.4309	1	0.97137	0.012615	1	1	0
ECM-receptor interaction	89	5.6547	2	0.97991	0.0088158	1	1	0.22222
Tryptophan metabolism	124	7.8784	3	0.98735	0.0055267	1	1	0.176
Ubiquinone and other terpenoid-quinone biosynthesis	103	6.5441	2	0.99096	0.0039423	1	1	0.10465
Systemic lupus erythematosus	136	8.6408	3	0.99327	0.002934	1	1	0.019231
Folate biosynthesis	83	5.2734	1	0.99578	0.0018349	1	1	0.022222
Bile secretion	246	15.63	7	0.99615	0.0016751	1	1	0.013889
N-Glycan biosynthesis	88	5.5911	1	0.99697	0.0013173	1	1	0.088608
Neuroactive ligand-receptor interaction	392	24.906	13	0.99771	0.00099443	1	1	0.073034
Porphyrin and chlorophyll metabolism	184	11.691	4	0.9978	0.00095561	1	1	0.12963
Arachidonic acid metabolism	138	8.7679	1	0.99989	4.77E-05	1	1	0.2437
Steroid hormone biosynthesis	159	10.102	1	0.99997	1.18E-05	1	1	0.10329
Drug metabolism - cytochrome P450	172	10.928	1	0.99999	4.96E-06	1	1	0.026846
Chemical carcinogenesis	181	11.5	1	0.99999	2.72E-06	1	1	0.026144
Olfactory transduction	456	28.972	5	1	1.16E-09	1	1	0.32558
Metabolism of xenobiotics by cytochrome P450	197	12.516	1	1	9.35E-07	1	1	0.30583
