# Supplementary material for: Oncogenic Integration of Nucleotide Metabolism via Fatty Acid Synthase in Non-Hodgkin Lymphoma
Source: Front Oncol. 2021 Oct 26;11:725137. doi: 10.3389/fonc.2021.725137 (PMC8576537; doi:10.3389/fonc.2021.725137)
Supplement: Supplementary Table S2 — Concentration of nucleotide pools in cerulenin treated bNHL cells. Quantitative mass spectrometry analysis of cerulenin treated bNHL cells, normalized per million cells, represented in μM or *ion counts. [file DataSheet_3.pdf]

Table S2: Concentration of nucelotides in cerulenin treated bNHL cells

Metabolites	Treatment	Primary DLBCL		Raji		SUDHL4		SUDHL10	
		Concentration	*P value*	Concentration	*P value*	Concentration	*P value*	Concentration	*P value*
**AMP**	Control	1420 ± 105	*0.0023*	32847 ± 145	*>0.0001*	8179 ± 557	*0.0169*	12629 ± 662	*>0.0001*
	Cerulenin	737 ± 136		11432 ± 673		6870 ± 114		*723 ± 42*	
**GMP**	Control	6.6 ± 0.5	*0.0011*	19.5 ± 0.9	*>0.0001*	3.5 ± 0.1	*0.0122*	5.7 ± 0.1	*>0.0001*
	Cerulenin	2.9 ± 0.5		5.9 ± 1		4.4 ± 0.34		0.2 ± 0.07	
**UMP**	Control	6.7 ± 0.7	*0.0003*	13.3 ± 0.5	*>0.0001*	4.4 ± 0.2	*>0.0001*	8.9 ± 0.2	*>0.0001*
	Cerulenin	1.9 ± 0.1		5.3 ± 0.1		1.4 ± 0.05		0.1 ± 0.01	
**CMP**	Control	2.0 + 0.3	*0.0261*	4.2 ± 0.1	*0.0001*	1.36 ± 0.06	*0.0005*	2.2 ± 0.1	*>0.0001*
	Cerulenin	0.9 ± 0.4		2.3 ± 0.2		0.5 ± 0.1		0.06 ± 0.01	
**ATP**	Control	728 ± 63	*0.0397*	246 ± 7	*0.0069*	177 ± 3.3	*0.0001*	191 ± 20	*0.0119*
	Cerulenin	618 ± 3		394 ± 40		99 ± 3.9		107 ± 4	
**GDP**	Control	26 ± 1.6	*0.0078*	35 ± 4	*0.0062*	13.9 ± 0.3	*0.0001*	16 ± 1.6	*0.0001*
	Cerulenin	7.4 ± 6.3		21 ± 1.5		8.5 ± 0.5		1.6 ± 0.8	
**UTP**	Control	256 ± 19	*0.0015*	58 ± 3.7	*>0.0001*	50 ± 0.7	*>0.0001*	82 ± 1.5	*>0.0001*
	Cerulenin	156 ± 10		113 ± 2.5		9 ± 0.6		31 ± 1	
**CTP**	Control	42 ± 4.4	*NS*	10 ± 0.54	*>0.0001*	8.6 ± 1.2	*0.0008*	10 ± 0.8	*0.0027*
	Cerulenin	31 ± 4.9		26 ¨ 0.8		1.4 ± 0.69		7.5 ± 0.1	
**6-PG***	Control	16341 ± 2749	*0.0032*	316 ± 548	*0.0013*	0	*NS*	0	*>0.0001*
	Cerulenin	38657 ± 5487		14452 ± 3019		647 ± 564		11665 ± 602	
**AICAR***	Control	26877 ± 5601	*0.0011*	242 ± 420	*>0.0001*	503 ± 81	*0.0004*	19324 ± 389	*>0.0001*
	Cerulenin	0		60072 ± 3286		0		345 ± 300	

Concentrations in μM normalized per million cells, * ion counts normalized per million cells
